# GluOC promotes proliferation and metastasis of TNBC through the ROCK1 signaling pathway

**DOI:** 10.1186/s12935-024-03445-8

**Published:** 2024-07-25

**Authors:** Jiaojiao Xu, Keting Dong, Xue Bai, Miao Zhang, Qian Du, Lei Chen, Jianhong Yang

**Affiliations:** Medical School, University of Chinese Academy of Sciences, Beijing, 101400 China

**Keywords:** TNBC, Osteocalcin, ROCK1, Proliferation, Metastasis

## Abstract

**Background:**

Triple negative breast cancer (TNBC) is a type of breast cancer that is negative for oestrogen receptor, progesterone receptor and human epidermal growth factor receptor 2, is highly malignant and aggressive, lacks of corresponding targeted therapy, and has a relatively poor prognosis. Therefore, understanding the mechanism of TNBC development and formulating effective treatment strategies for inducing cell death are still urgent tasks in the treatment of TNBC. Research has shown that uncarboxylated osteocalcin can promote the proliferation of prostate cancer, lung adenocarcinoma and TNBC cells, but the mechanism by which GluOC affects TNBC growth and metastasis needs further study.

**Methods:**

MDA-MB-231 breast cancer cells were used for in vitro cell analysis. Key target molecules or pathways were identified by RNA sequencing, and migration ability was detected by scratch assays, Transwell assays, cell adhesion assays and western blot analysis. Fluorescence staining, colony detection, qRT‒PCR and flow cytometry were used to detect apoptosis, oxidative stress, the cell cycle and the stemness of cancer cells, and a xenotransplantation model in BALB/C nude mice was used for in vivo analysis.

**Results:**

This study demonstrated that GluOC facilitates the migration of MDA-MB-231 breast cancer cells through the ROCK1/MYPT1/MLC2 signalling pathway and promotes the proliferation of TNBC cells via the ROCK1/JAK2/PIK3CA/AKT signalling pathway. Experiments in nude mice demonstrated that GluOC promoted tumour cell proliferation and metastasis in tumour-bearing mice, which further clarified the molecular mechanism of TNBC growth and invasion.

**Conclusion:**

Our findings highlight the importance of GluOC in driving TNBC progression and its association with poor patient outcomes. This study clarifies the functional effects of GluOC on TNBC growth, providing insight into the molecular basis of TNBC and potentially providing new ideas for developing targeted therapies to improve patient outcomes.

**Supplementary Information:**

The online version contains supplementary material available at 10.1186/s12935-024-03445-8.

## Background

The World Health Organization’s International Agency for Research on Cancer (IARC) 2018 GLOBOCAN report revealed that breast cancer surpassed lung cancer as the most common cancer among women, with approximately 20,000 cases per year [1]. Triple negative breast cancer (TNBC) is highly aggressive, accounting for 10-20% of total cases [2–4]. TNBC is a breast cancer subtype that lacks progesterone receptor (PR), and estrogen receptor (ER), expression and lacks epidermal growth factor receptor 2 (HER2) protein overexpression [5]. TNBC is more likely to occur in young women than in other populations, disease progression is rapid, the relapse rete is high, and the prognosis is poor. [6]. Compared with other types of breast cancer, TNBC lacks effective molecular targets and is prone to metastasis [7]. The median overall survival of patients with TNBC metastasis is approximately 18 months, which is much lower than that of patients with other breast cancer subtypes, whose survival can exceed five years [8]. These studies suggest an urgent need to explore new therapeutic targets for TNBC.

Osteocalcin is the most abundant noncollagen protein in bones [9]. It is one of the indicators of bone metabolism, and most of it is deposited in the bone matrix [10, 11].When bone resorption occurs, the acidic environment created by osteoclasts located in the resorptive lacunar can decarboxylate osteocalcin present in the extracellular matrix, allowing large amounts of uncarboxylated osteocalcin (GluOC) to be released into the bloodstream [12, 13]. GluOC is believed to act as a hormone, regulate glucose metabolism, promote testosterone secretion, act on muscle tissue to improve muscle sensitivity to insulin, and improve cognitive function [11]. In addition, a link has been noted between osteocalcin and tumourigenesis [14]. Ye and Kayed et al. revealed that osteocalcin can promote the proliferation of PC-3 human prostate cancer cells and pancreatic cancer cells. [15, 16]. Osteocalcin production is associated with prostate cancer metastasis [17]. Osteocalcin can accumulate in neutrophils to promote the development of lung cancer [18] and facilitate the development of prostate cancer cells through Group C6 member A of the G protein-coupled receptor family (GPRC6A) receptor [15]. Studies by Pietschmann et al. and Salem et al. showed that serum osteocalcin levels were markedly greater in patients with breast cancer and bone metastases than in healthy controls [19]. These studies provide new insights into the effects of GluOC on tumourigenesis, suggesting that osteocalcin and GluOC may be useful targets for the prevention of bone metastasis in breast cancer. Our previous study revealed that GluOC promotes TNBC cell proliferation and migration, but the underlying still needs to be further explored [20].

Rho-associated spiral kinases (ROCK) mediate many pathophysiological signals and play a variety of biological roles through binding with downstream target effectors, including those involved in contraction, adhesion, migration, proliferation, and apoptosis [21]. ROCK1 plays vital roles in the proliferation, migration and invasion of TNBC [22–25]. In addition, there is growing evidence that signalling pathways, including the PI3K/AKT [26], JAK/STAT [27], Wnt/beta-catenin [28], and Hedgehog signalling pathways [29], play crucial roles in cancers. The PI3K/Akt pathway can regulate tumour proliferation and survival, and plays an important role in the progression of breast cancer. PI3K gene alterations occur in nearly 30% of TNBC patients [30, 31]. Studies have shown that mutations in the P3-α gene PIK110CA account for 20-25% of PI3K family mutations [32]. Moreover, these mutations can affect the proliferation and metastasis of TNBC, and can be used as a potential therapeutic target for TNBC therapy. However, whether GluOC can activate ROCK1 and the PI3K/AKT signalling pathway to promote TNBC proliferation and metastasis has not been reported.

In this study, we performed transcriptomics analysis to identify the genes associated with GluOC-mediated promotion of MDA-MB-231 breast cancer cell proliferation and migration for further study. The results showed that GluOC directly affected the migration of MDA-MB-231 breast cancer cells via the ROCK1/MYPT1/MLC2 signalling pathway. Furthermore, we found that ROCK1 plays a key role in the proliferation of TNBC cells. Therefore, we treated MDA-MB-231 breast cancer cells with the ROCK1 inhibitor Y-27632 and/or GluOC. The results showed that GluOC inhibited the apoptosis of cancer cells via the ROCK1/JAK2/PIK3CA/AKT signalling pathway, promoted cell cycle progression, reduced ROS levels during cancer cell death, and promoted an increase in stem cell properties. Studies in nude mice also confirmed that GluOC promoted the proliferation and metastasis of TNBC cells.

## Materials and methods

### Transcriptomic analysis

MDA-MB-231 breast cancer cells were divided into con and 160 ng/mL GluOC treatment groups. The cells were collected for omics analysis, which was performed at Shanghai Applied Protein Technology Co., Ltd. (Shanghai, China). The detailed information is provided in the supplemental information.

### Wound healing

The migration ability of cancer cells was calculated by wound healing experiments. When the density of cancer cells in the six-well plate reached approximately 90%, the cell monolayer was scraped with a 10 µl pipette tip, and the fragments were washed three times with PBS. The cells were incubated in medium supplemented with Y-27632 (MCE, HY-10071) and/or GluOC for 24 h and observed under an inverted light microscope (Bio-Rad, 1450031). Cell images were taken at 20×magnifcation. Three regions with cells were randomly selected to assess cell migration. The wound area was calculated using ImageJ software version 6 (National Institutes of Health). Wound closure %: (A_t=0 h_ – A_t=24 h_)/A_t=0 h_×100%. All experimental data are from three independent experiments.

### Cell migration assay

A transwell chamber (diameter 6.5 mm, aperture 8 µM, Corning Costar, Cambridge, MA) was used to detect cell migration. After starvation treatment for 12 h, the cells were washed three times with PBS and subsequently resuspended in the upper chamber of the transwell chamber. The cell density in the upper chamber was 1 × 10^3^ cells/well. Then, 600 µl of medium containing 10% foetal bovine serum (FBS, VivaCell, C04001-500) was added to the lower chamber. The 24-well plates were cultured in an incubator for 8 h and then cultured for 24 h after the addition of Y-27632 and GluOC. After washing with PBS, the transwell chamber was fixed with 10% formaldehyde solution for 30 min, stained with 1% crystal violet solution (Solarbio, #G1064), and cultured for 15 min. After the chamber was dry, the cells above the chamber were wiped and photographed with an inverted microscope (Leica, DM1750). Cell images were taken at 40× magnification.

### Cell adhesion assay

For cell adhesion assays, the cells were incubated in 12-well plates (3 × 10^5^ per well) for 10–180 s. Y-27632 and GluOC were added to 12-well plates and incubated for 24 h. Then, the cells in each well were trypsinized (1 ml of trypsin was diluted in 19 ml of PBS) for various lengths of time. At 10 s, 60 s, 120 s, 180 s and 240 s, the adherent cells were gently washed with PBS, trypsinized and counted. Each experiment was repeated three times.

### Colony formation assay

MDA-MB-231 breast cancer cells were digested with trypsin and then resuspended in a 24-well plate at a density of 100 cells/well. The plates were then incubated with Y-27632 and GluOC for 24 h. Subsequently, the waste liquid was discarded every 2 days, and the medium was replaced with fresh medium for 14 days. Finally, after washing with PBS 3 times, the cells were fixed with methanol, stained with 1% crystal violet solution for approximately 15 min, observed under an inverted microscope and photographed.

### Fluorescence staining

MDA-MB-231 breast cancer cells (3 × 10^4^) were inoculated in 6-well plates for incubation, and the stock solution was removed and added to Y-27632 and/or GluOC for incubation for 24 h. Then the cells were washed twice with precooled PBS and then fixed with paraformaldehyde solution at room temperature for 30 min. Then, 0.5 mL of Hoechst 33258 staining solution (10 µg/mL, Solarbio, C0021) was added to each well, and the plates were incubated at room temperature for 15 min in the dark. Then, the dye solution was removed, and the cells were washed with PBS 3 times for 3 min each. Finally, the 6-well plates were placed under a fluorescence microscope (Leica, DM6B) to observe the morphology of the cell nucleus.

### Apoptosis assay

MDA-MB-231 breast cancer cells were treated with Y-27632 or GluOC for 24 h, and the cells were harvested after washing with PBS 3 times. The cells were digested with pancreatic enzyme without EDTA for 10 min, 1 ml of culture solution was added, and the mixture was centrifuged at 1000 rpm for 5 min. The waste solution was discarded, and 1 ml of precooled PBS was added. The cells were precipitated again, the waste solution was discarded, 500 µL of 1x binding buffer was added, and the mixture was then suspended in 5 ml flow tube after filtration through a 70 μm filter membrane. Then, 5 µL of Annexin V/FITC was added, the solution was mixed (Solarbio, CA1020), and incubated at 37 ℃ in the dark for 5 min; 5 µL of propyl iodide solution (PI) was added, the mixture was incubated at 37 ℃ in the dark for 5 min, and the mixture was finally stored in ice in dark. The fluorescence intensity was measured by FACSCanto II flow cytometry (FACSAria™ III flow cytometer, Becton Dickinson and Company), and the proportions of apoptotic cells and dead cells were analysed by FlowJo software.

### Cell-cycle analysis

MDA-MB-231 breast cancer cells were inoculated into 6-well plates (3 × 10^4^ cells/well) and treated with Y-27632 and/or GluOC after adhesion. The cells were collected into a centrifuge tube, washed twice with PBS, 70% ethanol was added, and the tube was placed at 4 ℃ overnight for fixation. After the fixing solution was removed, the cells were washed with precooled PBS 3 times, resuspended in the prepared propyl iodide dyeing solution (Beyotime, C1052), incubated at 37 ℃ for 30 min in the dark, and then placed on ice for preservation. Finally, the fluorescence intensity of the PI-DNA complex in the cell suspension was measured using FACSCanto II flow cytometry (FACSAria™III flow cytometry, Becton Dickinson and Company), and the data were analysed using FlowJo software.

### Detection of reactive oxygen species (ROS)

MDA-MB-231 breast cancer cells were incubated with Y-27632 and/or GluOC for 24 h. After the cells were washed with precooled PBS for 2 times, they were incubated at 37 ℃ with 10 µmol/L H2DCFH-DA (Solarbio, CA1410) for 30 min and mixed by inversion every 5 min, and then the stained cells were harvested and washed twice with cold PBS. Finally, the fluorescence induced by ROS production was analysed by FACSCanto II flow cytometry. FlowJo software was used to analyse the data.

### Measurement of SOD levels

After 24 h of treatment with GluOC and Y-27632, the cells were washed twice with PBS and incubated with extraction reagents from a superoxide dismutase (SOD) WST-1 detection kit (Solarbio, BC5165); and the levels of SOD in the MDA-MB-231 cells were analysed according to the manufacturer’s instructions.

### Mitochondrial membrane potential (ΔΨm) detection

Breast cancer cells were treated with Y-27632 and/or GluOC for 24 h. After washing with PBS 2 times, 1 ml of cell culture medium was added, 1 ml of JC-1 (J22202, Lablead Biotech) working solution was added, the sample was thoroughly mixed, and the cells were incubated at 37 ℃ for 20 min. At the end of incubation, the waste solution was removed, and after washing with JC-1 staining buffer (1x) 5 times, 2 ml of cell culture solution was added, and the cells were observed under an inverted fluorescence microscope.

### Western blot analysis

MDA-MB-231 breast cancer cells were treated with Y-27632 and/or GluOC for 24 h, washed twice with prewarmed PBS, and lysed in a cocktail containing RIPA buffer for 10 min. The supernatant of the lysate was harvested by centrifugation at 12,000 rpm for 10 min at 4 ℃. The protein concentration was determined with a BCA protein assay kit (Lablead Biotech, B5001), and a known amount of bovine serum albumin (BSA) was used to standardize the protein concentration. Cell lysates containing the same amount of protein were subjected to SDS-PAGE, and the separated proteins were electrotransferred to PVDF membranes in a TransBolt-SD apparatus. At room temperature, the membrane was blocked in TBST with 5% no fat milk for 2 h and incubated overnight at 4 ℃ with diluted primary antibodies against β-actin (Cohesion, CPA9066), ROCK1 (Cell Signaling Technology, #4035), P-MYPT1 (Cell Signaling Technology, #5163), MYPT1 (Cell Signaling Technology, #2634), P-MLC2 (Cell Signaling Technology, #3671), MLC2 (Abmart, T55708), Bcl-2 (Cell Signaling Technology, #15,071), Bax (Cell Signaling Technology, #2772), cleaved-caspase3 (Wanleibio, WL01992), caspase3 (Cell Signaling Technology, #14,220), cleaved-caspase9 (Cell Signaling Technology, #9505T), caspase9 (Cell Signaling Technology, #9508), Nrf2 (Abmart, TA0639S), HO-1 (Wanleibio, WL02400), CDK1 (Abcam, ab13327), Cyclin A2 (Abcam, AB181591), Cyclin B1 (Cell Signaling Technology, #12,231), OCT4 (Wanleibio, WL03686), NANOG (Wanleibio, WL03273), SOX2 (Wanleibio, WL03767), P-JAK2 (Wanleibio, WL02997), JAK2 (Wanleibio, WL02188), P-PIK3CA (Bioss, bs-5570R), PIK3CA (Bioss, bs-2067R), P-AKT (Cell Signaling Technology, #4060), AKT (Abcam, 179,463), P-CREB (Abmart, TN23982), CREB (Cell Signaling Technology, #9197). After the membranes were washed with TBST for 30 min, they were incubated with a suitable horseradish peroxidase conjugated secondary antibody (goat anti-rabbit IgG(H + L)-HRP, Lablead Biotech; S0101, goat anti-mouse IgG(H + L)-HRP, Lablead Biotech; S0100) for 2 h at room temperature. Finally, an enhanced chemiluminescence (ECL, Bio-Rad, #170–5060) kit was used to detect protein signals, and the greyscale values of each band were quantified by ImageJ software.

### Quantitative realtime PCR

The cells were treated with Y-27632 and/or GluOC. Total RNA was extracted using an RNA Simple Total RNA Kit (cat. no. DP419, Tiangen Biotech, Co., Ltd.). The purity and quality of the RNA were examined by a NanoDrop2000 (Thermo Scientifc, Wilmington, DE). A total of 1 µg of RNA was reverse transcribed into cDNA using Hifair III 1st Strand cDNA Synthesis SuperMix for qPCR (11141ES60, Yeasen Biotechnology). Then, quantitative real-time PCR (qRT–PCR) was performed using a SYBR Green qPCR kit (AQ132-24, TransGen Biotech.). The thermal cycling conditions were 94° 30 s, followed by 40 cycles of 94° for 5 s, 55° for 15 s, and 72° for 30 s. Quantification was based on the 2^(-△△Ct) method. β-Actin served as a control, and the primer sequences are shown in Supplemental Table 1.

### Mouse xenograft model

Twenty-one nude mice (BALB/C, 6-week-old female) were purchased from Beijing Lank Biotechnology Co., Ltd. (Experimental Animal Production Licence No.: SCXK (Beijing) 2019-0010). Animal studies were carried out according to the Guide for the Care and Use of Laboratory Animals (Eighth edition, 2011) and with the approval of the Animal Care and Use Committee of the University of Chinese Academy of Sciences. The mice were divided into three groups with 7 mice in each group and were injected subcutaneously with 5 × 10^5^ MDA-MB-231 cells. The tumour size was measured and recorded with Vernier calliper. When the tumour size was 60–120 mm^3^, equal amounts of PBS, 1 ng of GluOC and 3 ng of GluOC were injected. When the maximum tumour volume was 2000 mm^3^, the mice were killed by cervical dislocation after orbital blood samples were obtained, and the tumour tissues and heart, liver, spleen, lung and kidney tissues were collected. The tissues were fixed with 4% paraformaldehyde (LEAGENE, DF0135) and frozen after dehydration for subsequent experiments. During subcutaneous injection, the cells were suspended in a mixture of 50 µl of PBS and Matrigel (1:1, v/v) and implanted into a subcutaneous fat pad (5 × 10^5^ cells in MDA-MB-231) of 6-week-old female BALB/c nude mice. Tumour size was measured every 2 to 3 days to monitor tumour growth. The tumour volume was calculated as follows: V (volume) = L (length)×W (width)^2^ × 0.5.

### Serum osteocalcin detection

Blood was taken from the eyeballs of the tumour-bearing mice, kept overnight at 4 ℃, and then centrifuged at 1,500×g for 20 min. Freshly prepared serum was stored at -80 ℃ for later use. Serum osteocalcin levels were detected in different groups of tumour-bearing mice with an ELISA kit (Cloud-Clone Corp, SEA471Mu).

### ALT/AST detection

Serum samples were collected from the tumour-bearing mice, and the degree of liver damage in the mice in the different groups was detected according to the instructions of the ALT/GPT (Nanjing Jiancheng Bioengineering Institute, C009-2-1) test instructions and the AST/GOT (Nanjing Jiancheng Bioengineering Institute, C010-2-1) test instructions.

### Immunohistochemistry (IHC) analysis

Mouse tumour tissue and heart, liver, spleen, lung and kidney organs were fixed with 4% paraformaldehyde, dehydrated with 30% sucrose for 12 h, embedded and cut into 6 μm slices. After antigen repair, peroxidase activity was quenched, the sections were blocked with 10% goat serum at 37℃ for 30 min and then treated with anti-Ki67 (Cell Signaling Technology, #12202) at 4 ℃ overnight. The membranes were then incubated with HRP-conjugated secondary antibodies, and the colour was developed with diaminobenzidine (DAB, Solarbio, DA1010). Finally, glycerin gelatine tablets were used and observed under a microscope.

### Haematoxylin and eosin (HE) staining

The tissue was first fixed in 4% paraformaldehyde for 72 h, followed by dehydration with 30% sucrose. The tissue was then encased in optimal cutting temperature compound (OCT, SAKURA, 4583) and cut into 6 μm slices. After the sections were stained with haematoxylin dye for 30 s, they were rinsed with running water for 2 min; stained with eosin dye for 2 min; soaked in 95% alcohol and anhydrous ethanol for 3 min and 5 min, respectively, for dehydration; cleared in xylene for 10 min; sealed with neutral resin; and photographed under a microscope.

### Masson staining

We prepared 6 μm slices of immobilized and dehydrated mouse lung tissue for staining and evaluation of lung injury using Masson’s trichrome staining kit (Solarbio, G1340). Sections were stained with Weigert iron haematoxylin solution for 5 min, differentiated with ethanol solution for 10 s, and then soaked in pure water. Then Masson’s blue solution was used to reverse bluing for 3 min and soak in pure water for 1 min. After staining for 5 min with Ponceau fuchsin staining solution, the samples were soaked in the corresponding weak acid working solution for 1 min. Then, the phosphomolybdic acid solution was added for 2 min, and the weak acid working solution was added for 1 min. The treated sections were directly put into aniline blue dyeing solution for 1 min and then soaked in weak acid working solution for 1 min. Glycerin gelatine tablets were then used and observed under a microscope.

### Statistical analysis

The data are presented as the mean ± standard deviation (SD). GraphPad Prism 9.4.0 software was used to perform the statistical analysis. The data are presented as the mean ± SD unless otherwise specified. P values were obtained by unpaired Student’s t–test, and *p* ≤ 0.05 was considered to indicate statistical significance. The in vitro experiments were repeated independently three times with consistent results.

## Results

### Systematic investigation of the transcriptional changes caused by GluOC

**Fig. 1:**
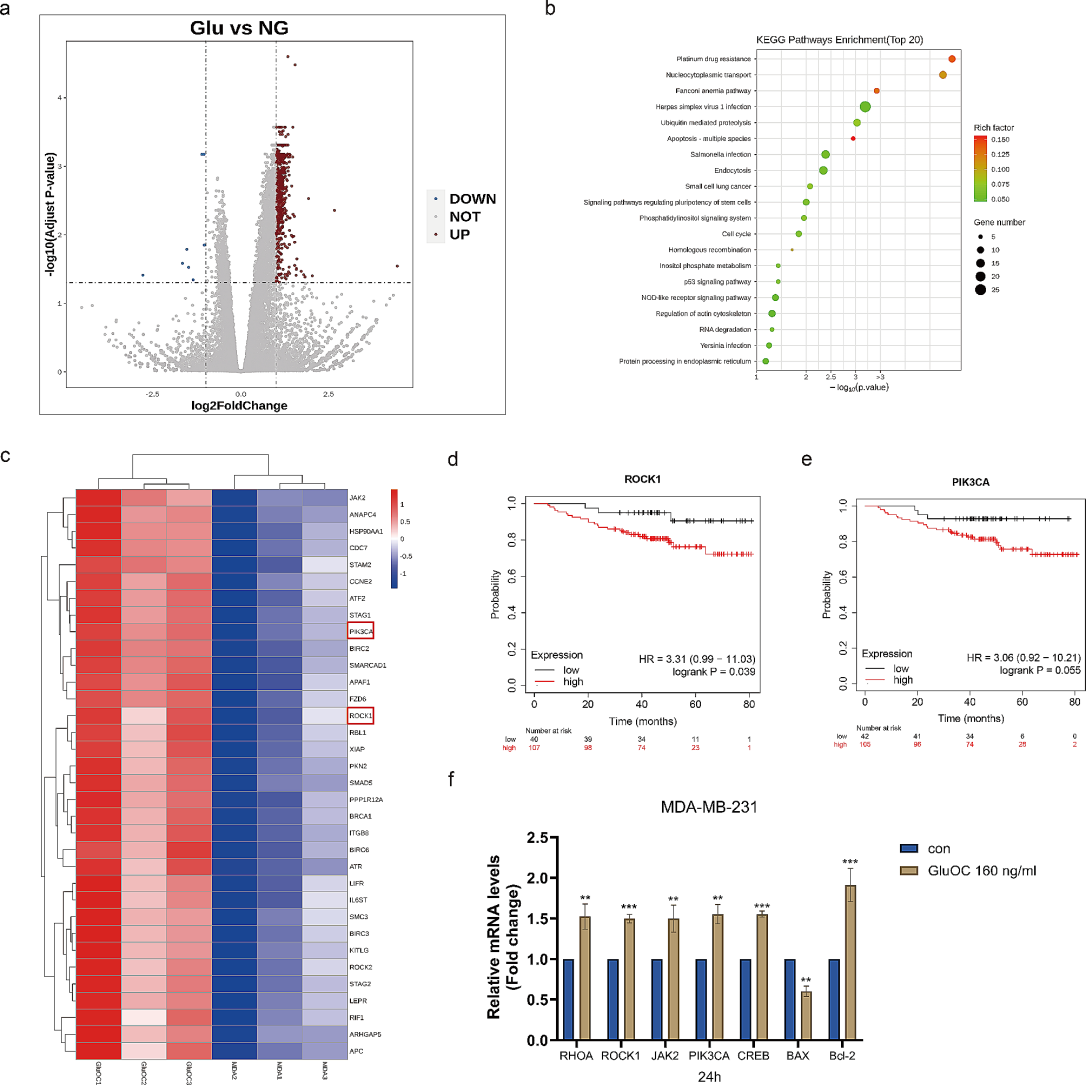
(**a**) DEGs were identified in the GEO dataset, and volcano maps of differentially expressed genes were generated via combined analysis of three GEO datasets. The red and blue dots represent the up- and down-regulated DEGs, respectively, while the grey dots represent genes whose expression did not significantly change. (**b**) KEGG pathway enrichment results of upregulated genes. The functional enrichment results were derived from the analysis, where different colours represent the significance of differential enrichment results. The larger the value is the smaller the FDR value, and the larger the number of enriched genes is the larger the circle. In the enrichment results, *P* < 0.05 or FDR < 0.05 was considered to indicate significant enrichment of a pathway. (**c**) Hierarchical clustering analysis and heatmap of the differentially expressed genes. (**d**-**e**) The associations of ROCK1 and PIK3CA with OS were analysed via the Kaplan–Mei plotter website. (**f**) qRT–PCR analysis of selected genes in MDA-MB-231 cells treated with 160 ng/mL GluOC for 24 h. The results represent at least three independent experiments, and the data are presented as the mean ± SD (*n* = 3). ^*^p < 0.05, ^**^p < 0.01, ^***^p < 0.001 compared with the control group

We divided MDA-MB-231 breast cancer cells into a control group and a 160 ng/mL GluOC-treated group and then performed transcriptomic analysis. By transcriptomic analysis, we found 597 upregulated differentially expressed genes (DEGs) and 8 downregulated DEGs (multiploidy logFoldChange ≥ 2 and P value < 0.05) (Fig. 1a). The enrichment of KEGG pathways indicated that many metabolic pathways, such as apoptosis, the cell cycle, and the upregulation of cancer stem cell pluripotency, were affected (Fig. 1b). Comprehensive KEGG pathway analysis of the DEGs revealed that the ROCK1 and PIK3CA signalling pathways were closely related to the occurrence and development of breast cancer (Fig. 1c). Higher ROCK1 and PIK3CA levels in TNBC patients were correlated with shorter overall survival (OS) according to the Kaplan–Mei plotter website (Fig. 1d and e). Subsequently, we performed qRT–PCR for verification, and the results showed that after adding GluOC into MDA-MB-231 breast cancer cells, the levels of ROCK1, JAK2, PIK3CA, Bcl-2, CREB were significantly increased, while the expression levels of Bax were significantly decreased (Fig. 1f).

### GluOC promotes the migration of MDA-MB-231 breast cancer cells through the ROCK1/MYPT1/MLC signalling pathway

**Fig. 2:**
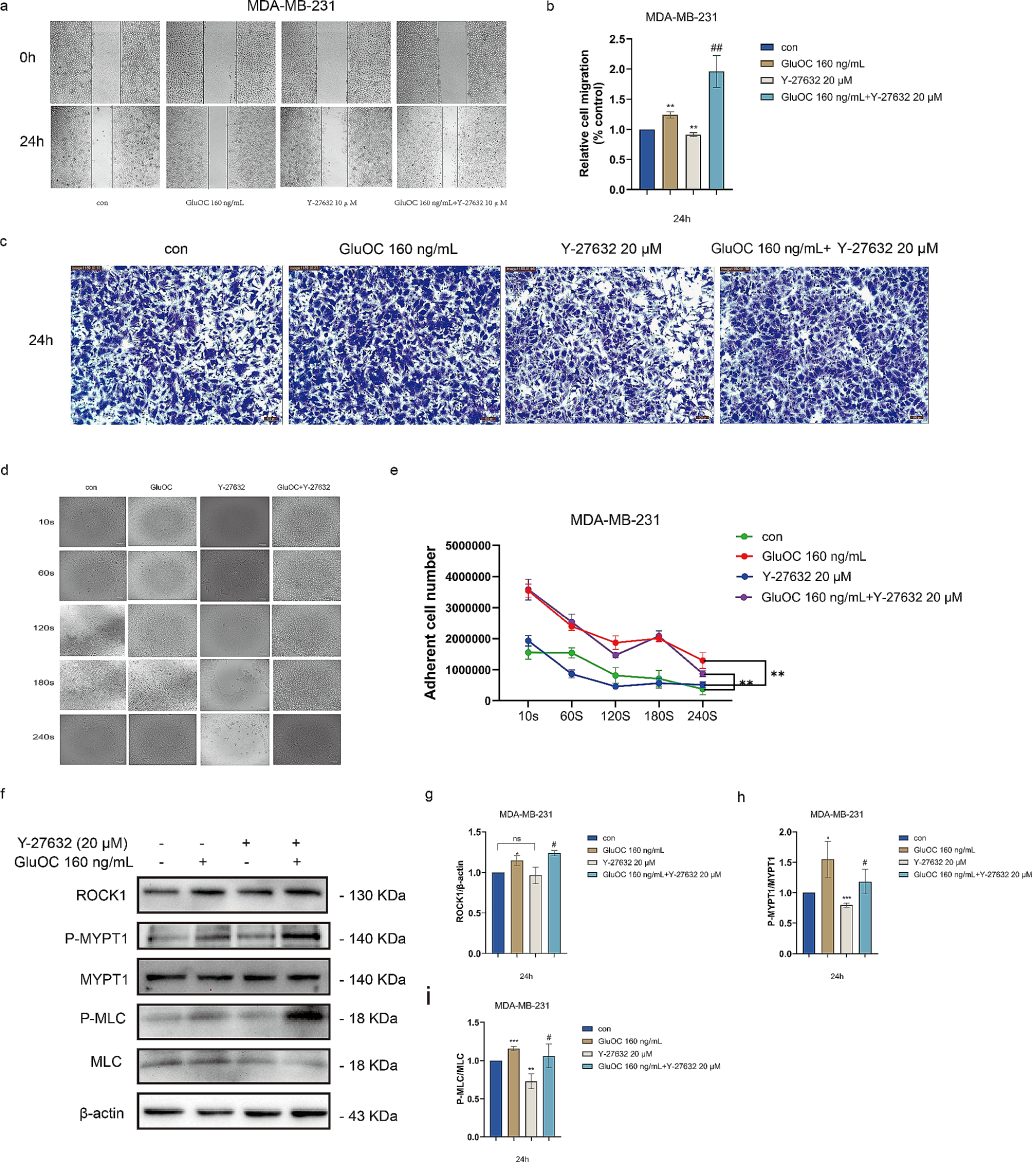
(**a**-**b**) For the wound healing assay MDA-MB-231 cells. Wound area before and after injury was measured to quantify the wound closure migration of MDA-MB-231cells at 20x magnification (% control). The results represent at least three independent experiments, and the data are presented as the mean ± SD (*n* = 3). (**c**) Transwell assays were used to determine the effect of ROCK1 inhibition on the migration ability of MDA-MB-231 cells. Photographs were taken with a microscope for counting at 40× magnification. (**d**-**e**) Cell attachment (sec) assay. (**f**-**i**) The protein levels of ROCK1, P-MYPT1 and P-MLC in MDA-MB-231 cells before and after treatment with GluOC and the ROCK1 inhibitor Y-27632 were determined by western blotting and quantified by densitometry with ImageJ software. β-Actin was used as the control. The results represent at least three independent experiments, and the data are presented as the mean ± SD (*n* = 3). ^*^p < 0.05, ^**^p < 0.01, ^***^p < 0.001 compared with the control group. ^#^*p* < 0.05, ^##^*p* < 0.01, ^###^*p* < 0.001 compared with the Y-27,632 group

GluOC significantly promoted the expression of the ROCK1 gene, which inhibited myosin phosphatase (MYPT1) through direct or indirect phosphorylation of myosin light chain (MLC), thus leading to actin-myosin contraction and subsequent invasion and metastasis of cells. According to the genomics results, the ROCK1 inhibitor Y-27632 was selected to explore whether GluOC promotes breast cancer metastasis through ROCK1. After treatment with GluOC, the results showed that GluOC increased the migration of MDA-MB-231 breast cancer cells and reduced the distance between the scratches under Y-27632 treatment conditions (Fig. 2a and b). Transwell assays also showed that after ROCK1 inhibition, the number of migrating cells decreased compared with that in the control group. GluOC significantly alleviated the inhibitory effect of Y-27632 on the migration ability of breast cancer cells, which manifested as an increase in the number of migrating cells (Fig. 2c). Additionally, cell adhesion experiments demonstrated that GluOC promoted the adhesion of MDA-MB-231 breast cancer cells and alleviated the decrease in cancer cell adhesion induced by Y-27632 (Fig. 2d and e). The above results indicated that GluOC promotes the migration of MDA-MB-231 breast cancer cells through ROCK1. Western blotting further verified that GluOC phosphorylates MYPT1 through ROCK1, which reduces the activity of MYPT1, thereby promoting the phosphorylation of MLC, and ultimately enhancing the migration of MDA-MB-231 breast cancer cells (Fig. 2f and i).

### GluOC inhibits apoptosis of MDA-MB-231 breast cancer cells through ROCK1

**Fig. 3:**
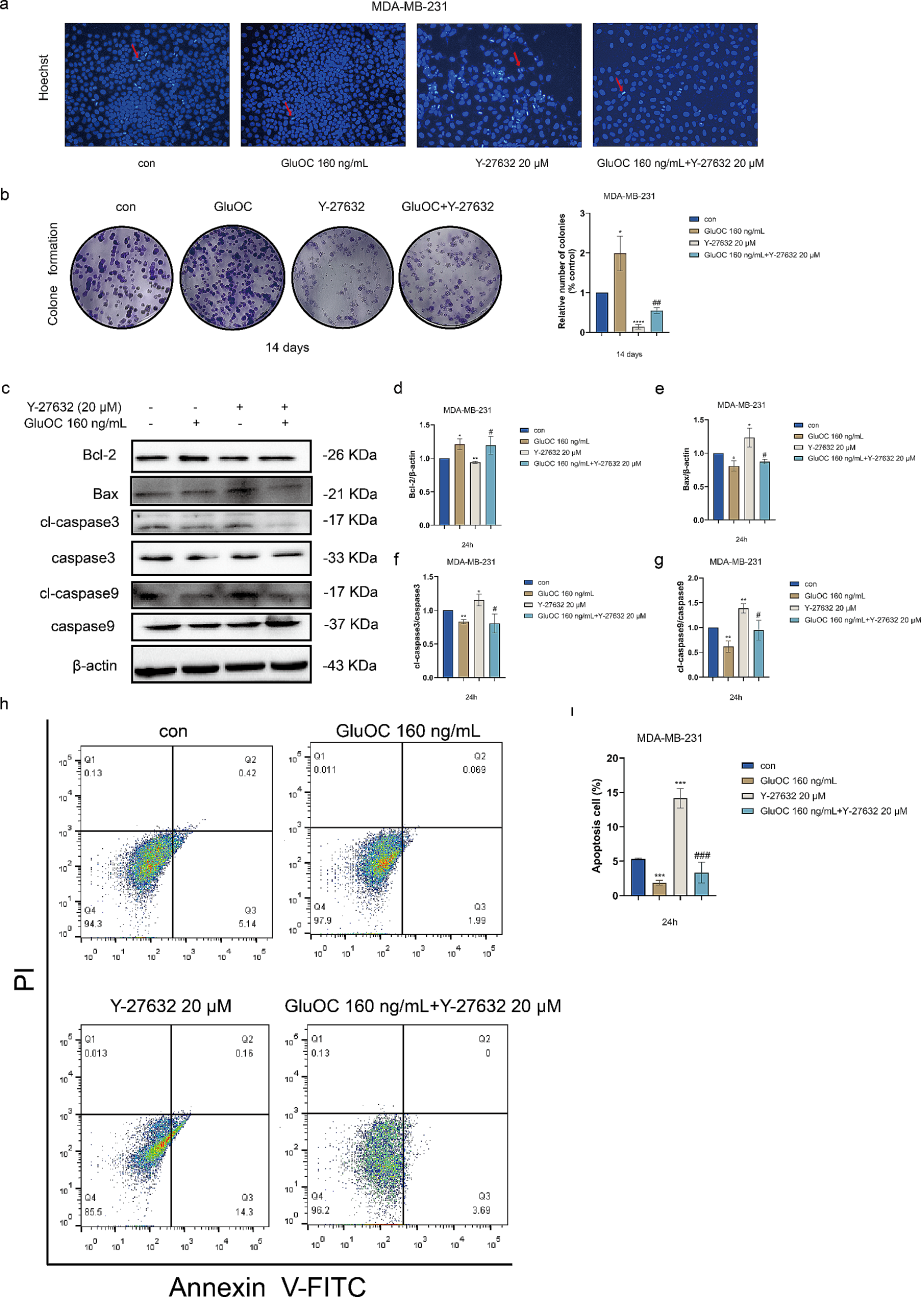
(**a**) Photomicrographs showing the images of MDA-MB-231 breast cancer cells subjected to Hoechst 33258 staining after treatment with GluOC and Y-27632 for 24 h. (**b**) Effect of GluOC on the colony formation of MDA-MB-231 breast cancer cells. (**c**-**g**) The protein expression levels of Bax, Bcl-2, caspase-3/9 and cleaved-caspase-3/9 in GluOC– and Y-27632–treated MDA-MB-231 breast cancer cells were determined by western blotting and quantified densitometrically with ImageJ software. β-Actin was used as the control. The results represent at least three independent experiments, and the data are presented as the mean ± SD (*n* = 3). The relative protein levels of Bax, Bcl-2, caspase-3/9 and cleaved-caspase-3/ 9 were statistically analysed. The protein levels were normalized to those in the control group. (**h**) The level of apoptosis of MDA-MB-231 breast cancer cells treated with GluOC and/or Y-27632 was assessed by Annexin V-FITC/PI double staining, and the control groups were untreated. (**i**) Statistical analysis of the apoptosis rate of MDA-MB-231 breast cancer cells treated with GluOC and/or Y-27632. ^*^p < 0.05, ^**^p < 0.01, ^***^p < 0.001 compared with the control group. ^#^*p* < 0.05, ^##^*p* < 0.01, ^###^*p* < 0.001 compared with the Y-27632 group

To explore the inhibition of MDA-MB-231 breast cancer cell apoptosis by GluOC via ROCK1, Hoechst 33258–stained nuclei were then examined by fluorescence microscopy. In the untreated cells and the Y-27632–treated cells, the nuclei exhibited dense blue foci, whereas GluOC–treated cells had a more regular and light blue shape, alleviating Y-27632–induced apoptosis (Fig. 3a). The colony formation assay results also showed that GluOC could increase colony formation in MDA-MB-231 breast cancer cells and attenuate cell damage caused by Y-27632 (Fig. 3b). The level of Bcl-2 in MDA-MB-231 breast cancer cells treated with GluOC was significantly increased, but Bax, cl-caspase3 and cl-caspase9 were significantly reduced, and GluOC blocked the decrease in Bcl-2 and increase in Bax, cl-caspase3 and cl-caspase9 induced by ROCK1 (Fig. 3c-g). Annexin V–FITC/PI double staining revealed that compared with those in the untreated group, the number of early apoptotic cells increased after Y-27632 treatment. After treatment with GluOC, the proportion of Annexin V–positive cells decreased compared with that in the Y-27632 group (Fig. 3h-i). The observation of GluOC–mediated attenuation of the increase in the percentage of apoptotic MDA-MB-231 breast cancer cells further supports the idea that GluOC can inhibit the apoptosis of MDA-MB-231 cells through ROCK1.

### GluOC inhibits oxidative stress in MDA-MB-231 breast cancer cells through ROCK1

**Fig. 4:**
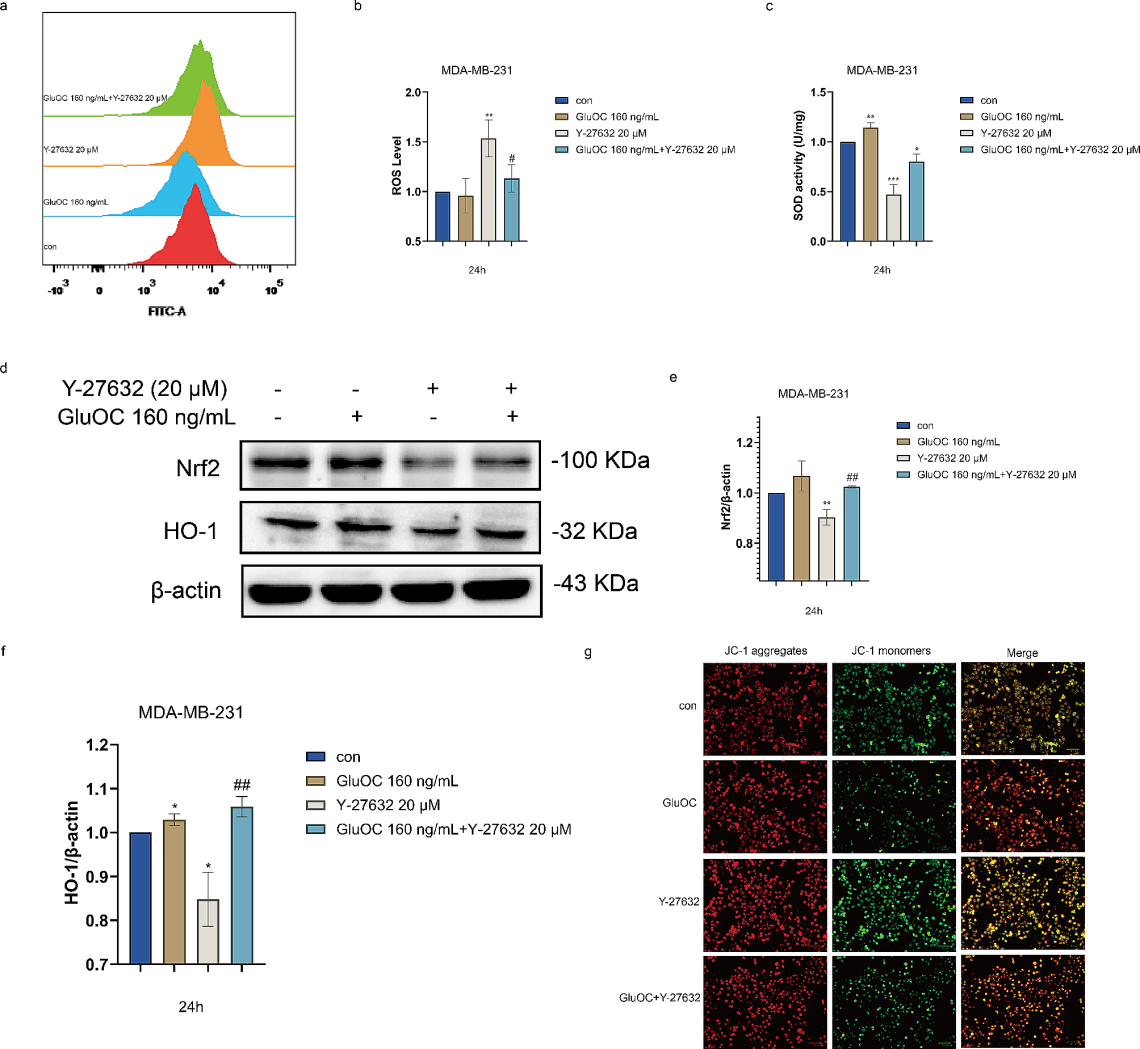
(**a**-**b**) ROS production in MDA-MB-231 breast cancer cells was detected by DCFDA after 24 h of treatment with Y-27632 and/or GluOC, and fluorescence was detected by flow cytometry. (c) SOD levels in cancer cells. (**d**-**f**) The protein expression levels of Nrf2 and HO-1 in GluOC– and Y-27632–treated MDA-MB-231 breast cancer cells were determined by western blotting and quantified by densitometry with ImageJ software. β-Actin was used as the control. The results represent at least three independent experiments, and the data are presented as the mean ± SD (*n* = 3). The relative protein levels of Nrf2 and HO-1 were statistically analysed. The protein levels were normalized to those in the control group. (**g**) MDA-MB-231 breast cancer cells were detected via the JC-1 assay. Cells were treated with the indicated concentrations of GluOC and/or Y-27632 and subsequently examined by fluorescence microscopy at 20× magnification. The results represent at least three independent experiments, and the data are presented as the mean ± SD (*n* = 3). ^*^p < 0.05, ^**^p < 0.01, ^***^p < 0.001. compared with the control group. ^#^*p* < 0.05, ^##^*p* < 0.01, ^###^*p* < 0.001 compared with the Y-27,632 group

Excess ROS activate different cell death pathways, such as apoptosis, which limits cancer progression. Therefore, we first used streaming to detect ROS levels. GluOC increased the antioxidant effect on cancer cells and alleviated Y-27632–induced oxidative stress (Fig. 4a and b). Similarly, we measured SOD levels inside the cells. The results showed that the addition of GluOC significantly increased the intracellular SOD content and attenuated the decrease inx intracellular SOD induced by the addition of Y-27632 (Fig. 4c). Subsequently, we detected the expression levels of the antioxidant factors Nrf2 and HO-1, and the results showed that GluOC alleviated the reduction in the Nrf2 and HO-1 protein levels caused by the addition of Y-27632 (Fig. 4d and f). These findings indicated that GluOC promotes the expression of Nrf2 and HO-1 through ROCK1, thereby increasing the antioxidant capacity of MDA-MB-231 cells and promoting the proliferation of cancer cells. We further used JC-1 staining to detect the mitochondrial membrane potential (MMP) of MDA-MB-231 breast cancer cells treated with GluOC. The fluorescence staining results showed that compared with that in the control group, the green fluorescence in the GluOC treatment group was significantly lower. However, the green fluorescence of cells in the Y-27632 group was significantly increased, and the GluOC attenuated the effects of Y-27632. This finding suggested that GluOC can reduce MMP damage of MDA-MB-231 breast cancer cells through ROCK1 (Fig. 4e). These results suggest that GluOC can inhibit ROS and MMP damage through ROCK1, thereby inhibiting cancer cell apoptosis.

## GluOC promotes the cycle progression of MDA-MB-231 breast cancer cells through ROCK1

**Fig. 5:**
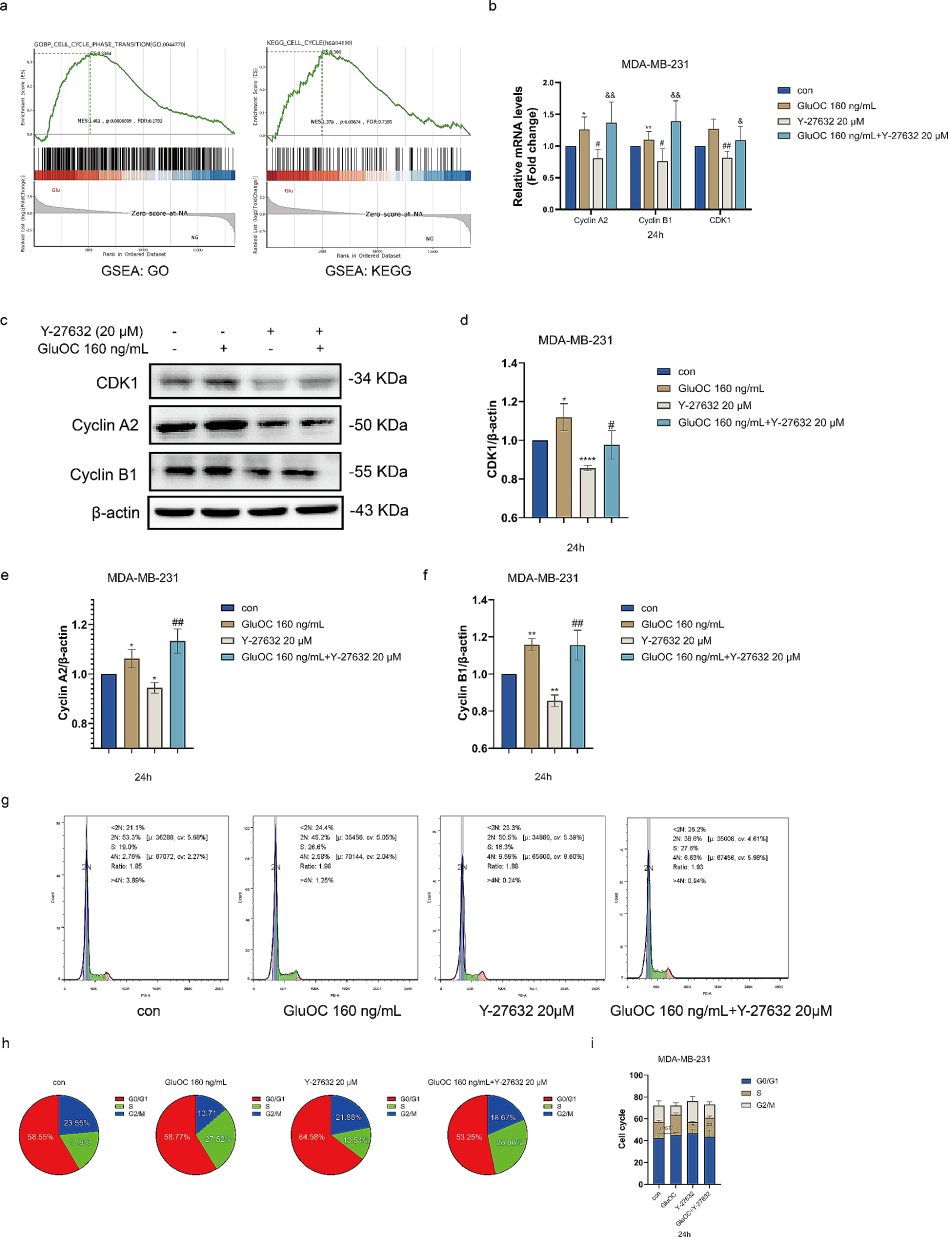
(**a**) GSEA of the dataset was performed, and the correlation enrichment map after GluOC treatment is shown. (**b**) GluOC promotes cell cycle progression through ROCK1. The gene expression of cyclin A2, cyclin B1 and CDK1 in MDA-MB-231 cells before and after GluOC and after stimulation with the ROCK1 inhibitor Y-27632 were determined by qRT–PCR. (**c**-**f**) The protein expression levels of cyclin A2, cyclin B1 and CDK1 in GluOC– and Y-27632–treated MDA-MB-231 breast cancer cells were determined by western blotting and quantified densitometrically with ImageJ software. β-Actin was used as the control. The results represent at least three independent experiments, and the data are presented as the mean ± SD (*n* = 3). The relative protein levels of cyclin A2, cyclin B1 and CDK1 were statistically analysed. The protein levels were normalized to those in the control group. (**g**) The cell cycle distribution of MDA-MB-231 breast cancer cells treated with GluOC and/or Y-27632 was detected by flow cytometry. (**h**-**i**) Statistical analysis of the percentages of MDA-MB-231 breast cancer cells in the G0/G1, S and G2/M phase. The results represent at least three independent experiments, and the data are presented as the mean ± SD (*n* = 3). ^*^p < 0.05, ^**^p < 0.01, ^***^p < 0.001 compared with the control group. ^#^*p* < 0.05, ^##^*p* < 0.01, ^###^*p* < 0.001 compared with the Y-27632 group

Gene set enrichment analysis (GSEA) consistently revealed that the addition of GluOC affected breast cancer cell cycle progression (Fig. 5a). qRT–PCR was used to detect the expression levels of cell cycle related genes in MDA-MB-231 breast cancer cells (Fig. 5b). The results showed that the expression levels of cyclin A2, cyclin B1 and CDK1 induced by Y-27632 decreased, and the effect of Y-27632 was reversed after GluOC was added (Fig. 5c and f). In addition, western blotting showed that GluOC upregulated the expression levels of cyclin A2, cyclin B1 and CDK1 through ROCK1. Then, we used PI staining to study the effect of GluOC on the cell cycle distribution of MDA-MB-231 breast cancer cells. Compared with that in the control group, the proportion of S-phase cells decreased significantly after 24 h of treatment with Y-27632, and the proportion of S-phase cells increased compared with that in the Y-27632 group after the addition of GluOC and Y-27632 at the same time point (Fig. 5g and i). The results showed that GluOC could reduce the inhibitory effect of Y-27632 on the S-phase progression of MDA-MB-231 breast cancer cells, promote cell cycle progression, and subsequently promote the proliferation of MDA-MB-231 breast cancer cells.

## GluOC enhances the stem cell properties of MDA-MB-231 breast cancer cells through ROCK1

**Fig. 6:**
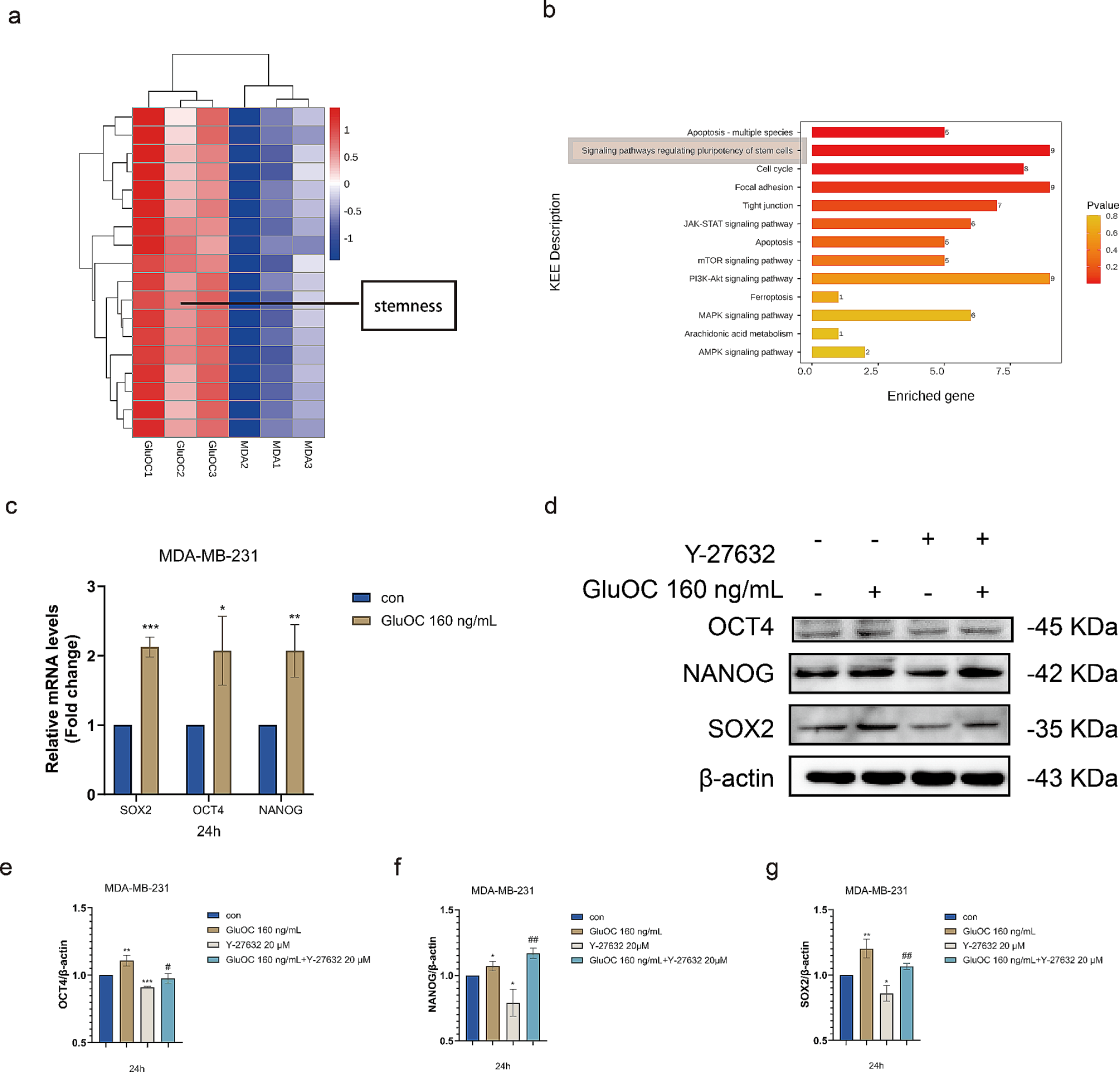
(**a**) Heatmap of the upregulated differentially expressed genes. (**b**) KEGG pathways enriched in the DEGs. (**c**) The gene expression levels of SOX2, OCT4 and NANOG in MDA-MB-231 cells before and after GluOC treatment were determined by qRT–PCR. (d-g) The protein expression levels of OCT4, NANOG and SOX2 in GluOC– and Y-27632–treated MDA-MB-231 breast cancer cells were determined by western blotting and quantified by densitometry with ImageJ software. β-Actin was used as the control. The results represent at least three independent experiments, and the data are presented as the mean ± SD (*n* = 3). The relative protein levels of OCT4, NANOG and SOX2 were statistically analysed. The protein levels were normalized to those in the control group. The results represent at least three independent experiments, and the data are presented as the mean ± SD (*n* = 3). ^*^p < 0.05, ^**^p < 0.01, ^***^p < 0.001 compared with the control group. ^#^*p* < 0.05, ^##^*p* < 0.01, ^###^*p* < 0.001 compared with the Y-27632 group

There is growing evidence that TNBC cells contain the highest proportion of cancer stem cells (CSCs). According to the DEG and KEGG, GluOC also plays a role in regulating stem cell pluripotency (Fig. 6a and b). Then, we used qRT–PCR to verify the expression levels of OCT4, NANOG and SOX2 at the transcriptional level. GluOC significantly promoted the expression of OCT4, NANOG, and SOX2 (Fig. 6c). Subsequently, we further verified whether ROCK1 was related to it, and the results showed that Y-27632 significantly inhibited the expression of OCT4, NANOG, and SOX2, while GluOC significantly attenuated the inhibitory effect of Y-27632 (Fig. 6d and g).

### GluOC promotes the proliferation of MDA-MB-231 breast cancer cells through the ROCK1/JAK2/PIK3CA/AKT signalling pathway

**Fig. 7:**
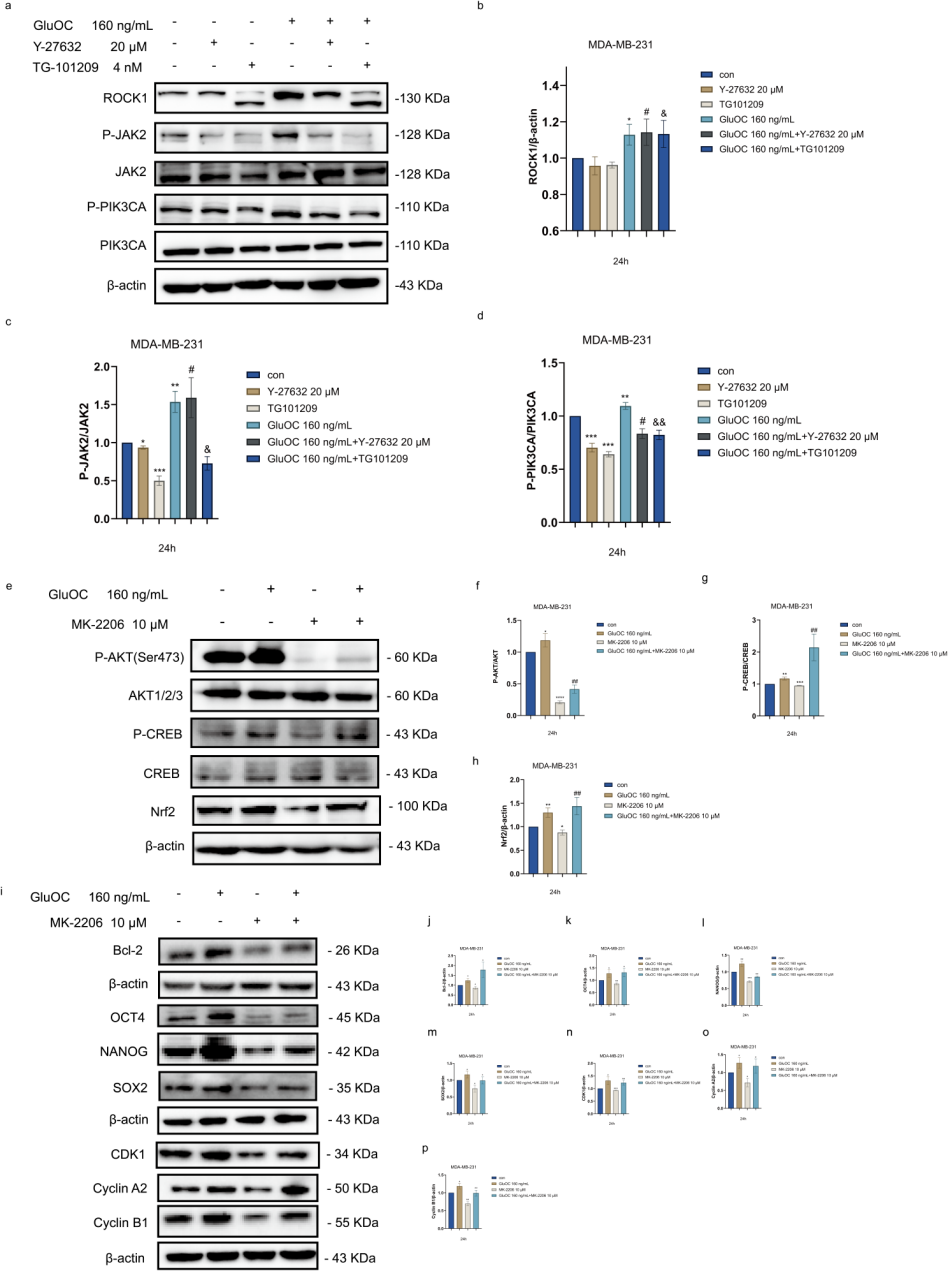
(**a**-**d**) The protein expression levels of ROCK1, JAK2, P-JAK2, PIK3CA and P-PIK3CA in GluOC–, Y-27632– and TG-101209–treated MDA-MB-231 breast cancer cells were determined by western blotting and quantified densitometrically with ImageJ software. β-Actin was used as the control. The results represent at least three independent experiments, and the data are presented as the mean ± SD (*n* = 3). ^*^p < 0.05, ^**^p < 0.01, ^***^p < 0.001 compared with the control group. ^#^*p* < 0.05, ^##^*p* < 0.01, ^###^*p* < 0.001 compared with the Y-27632 group. ^&^*p* < 0.05, ^&&^*p* < 0.01, ^&&&^*p* < 0.001 compared with the TG10129 group. (**e**-**h**) The relative protein levels of P-AKT, AKT, P-CREB, CREB and Nrf2 were statistically analysed by western blotting and quantified densitometrically with ImageJ software. β-Actin was used as the control. The results represent at least three independent experiments, and the data are presented as the mean ± SD (*n* = 3). ^*^p < 0.05, ^**^p < 0.01, ^***^p < 0.001 compared with the control group. ^#^*p* < 0.05, ^##^*p* < 0.01, ^###^*p* < 0.001 compared with the MK-2206 group. (i-p) The relative protein levels of Bcl-2, OCT4, NANOG, SOX2, CDK1, cyclin A2 and cyclin B1 were statistically analysed after the addition of GluOC and/or MK-2206. β-Actin was used as the control. The results represent at least three independent experiments, and the data are presented as the mean ± SD (*n* = 3). ^*^p < 0.05, ^**^p < 0.01, ^***^p < 0.001 compared with the control group. ^#^*p* < 0.05, ^##^*p* < 0.01, ^###^*p* < 0.001 compared with the MK-2206 group

PI3K/Akt plays an important role in cell regulation and is related to the cell cycle, differentiation, migration, proliferation and apoptosis. Using RNA-seq data, we further investigated the role of the ROCK1, JAK2 and PI3K/AKT pathways in GluOC–mediated regulation of TNBC. The results showed that ROCK1, P-JAK2 and P-PIK3CA protein levels were significantly greater in the GluOC group than in the control group. After the addition of Y-27632, the P-JAK2 and P-PIK3CA protein levels were decreased significantly. Compared with those in the Y-27632 inhibitor group, the P-JAK2 and P-PIK3CA protein levels were significantly increased after the addition of GluOC, and the results showed that ROCK1 regulated JAK2 and PIK3CA, thereby affecting the proliferation and apoptosis of MDA-MB-231 breast cancer cells. We also detected the protein expression levels of ROCK1, P-JAK2 and P-PIK3CA after the addition of the P-JAK2 inhibitor. The results showed that the protein expression level of ROCK1 did not change after the addition of the JAK2 inhibitor TG-101209, while the expression of P-PIK3CA decreased with decreasing P-JAK2 expression. Additionally, the addition of TG-101209 and GluOC attenuated the inhibitory effect of TG-101209, and the protein expression levels of P-JAK2 and P-PIK3CA were greater than those in the TG-101209 only group. We concluded that GluOC promotes the proliferation and apoptosis of MDA-MB-231 breast cancer cells through the ROCK1/JAK2/PIK3CA signalling pathway (Fig. 7a and d). In addition, we treated MDA-MB-231 breast cancer cells with GluOC and found that the protein expression levels of P-AKT, Nrf-2 and P-CREB were significantly greater than those in the control group. Compared with those in the control group, the protein expression levels of P-AKT, Nrf-2 and P-CREB in the MK-2206 treatment group were significantly decreased. GluOC reversed the inhibitory effect of MK-2206. In addition, we further examined the expression of MDA-MB-231 breast cancer cell proliferation, stemness markers and cell cycle–related proteins after the addition of MK-2206 and found that MK-2206 significantly decreased the expression of the Bcl-2, OCT4, NANOG, SOX2, CDK1, cyclin A2, and cyclin B1 proteins, while GluOC reversed the effect of MK-2206 (Fig. 7i and p). These results suggest that GluOC may promote the proliferation of MDA-MB-231 breast cancer cells through the ROCK1/JAK2/PI3K/AKT signalling pathway.

### GluOC promotes the growth of breast cancer in nude mice

**Fig. 8:**
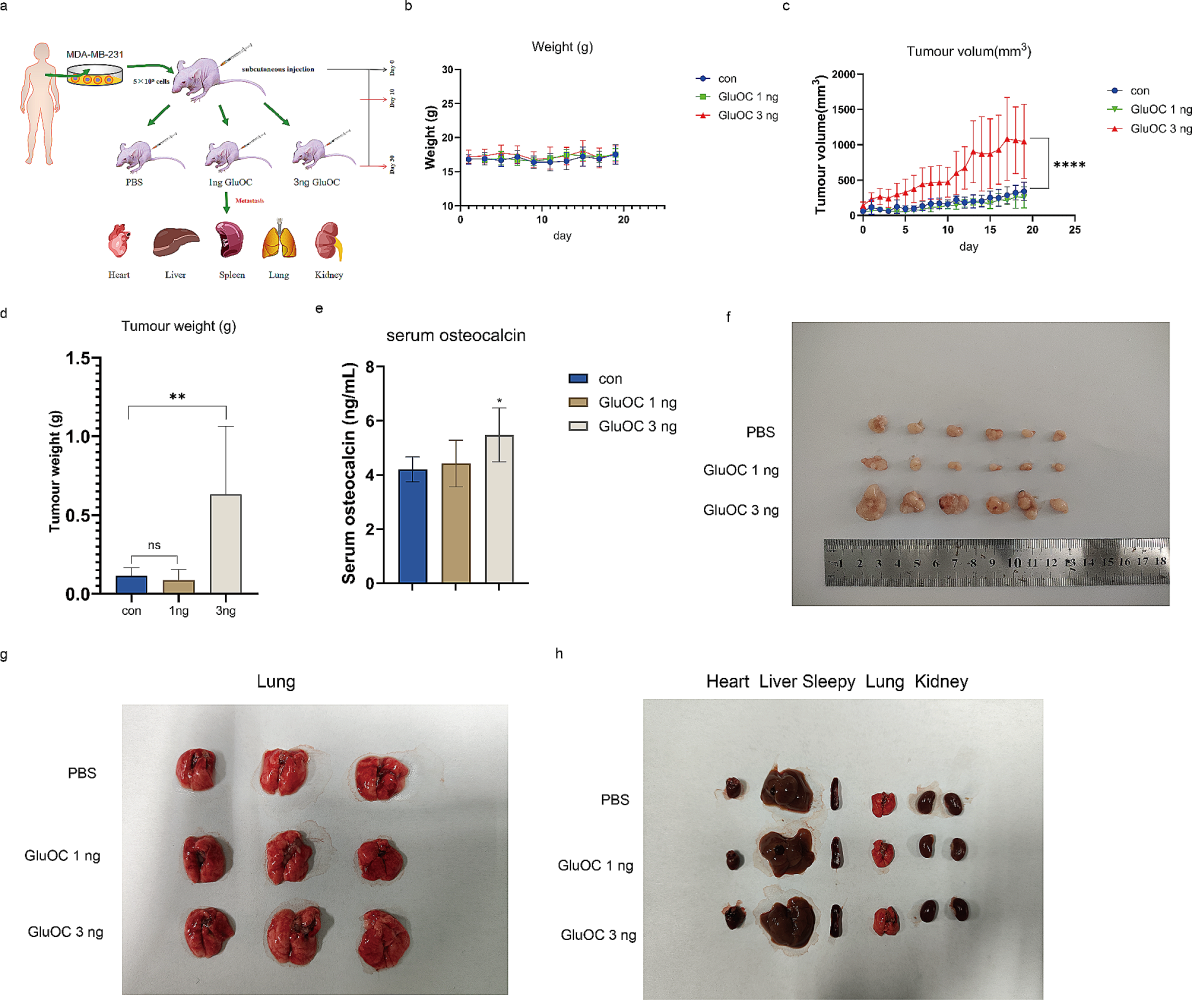
(**a**) Schematic flowchart of the in vivo MDA-MB-231 cell metastasis model. (**b**) Individual mouse growth curves are shown. (**c**) Average tumour average growth curve. (**d**) Statistics of tumour weight (*n* = 6). (**e**) Serum osteocalcin content of the mice (*n* = 5). (**f**) Image of tumours. (**g**) Representative images of whole lungs. (**h**) Heart, liver, spleen and kidney shape and size were dissected and observed. The results represent at least three independent experiments, and the data are presented as the mean ± SD (*n* = 6). ^*^p < 0.05, ^**^p < 0.01, ^***^p < 0.001 compared with the control group. ^#^*p* < 0.05, ^##^*p* < 0.01, ^###^*p* < 0.001 compared with the PBS group

Due to the ability of GluOC to promote the proliferation and migration of MDA-MB-231 breast cancer cells, we next investigated whether GluOC promotes tumour growth and metastasis in vivo (Fig. 8a). After tumour formation, 1 ng and 3 ng of GluOC were administered daily near the tumour, and the mice were sacrificed when the tumour volume reached 2000 mm^3^. We found no significant difference in body weight between the groups (Fig. 8b). As shown in Fig. 8c, 3 ng of GluOC significantly promoted tumour growth in mice. Compared with that in the PBS group, the tumour weight in the 3 ng GluOC group was significantly greater, while 1 ng GluOC had no significant effect on tumour growth **(**Fig. 8d). Then, we collected serum from the mice and tested it with an ELISA kit. The results showed that the serum osteocalcin level of the mice in the 3 ng GluOC group was significantly greater than that of the control group, which was consistent with the results of previous studies **(**Fig. 8e). After administration, the tumour volume in the 3 ng GluOC group was significantly greater than that in the control group **(**Fig. 8f). To determine whether GluOC affects the metastatic ability of MDA-MB-231 cells, we collected lung, heart, liver, spleen, lung and kidney tissues from mice after different treatments, and examined whether there was metastasis in the lungs by HE staining **(**Fig. 8g and h).

### GluOC promotes the metastasis of breast cancer in nude mice

**Fig. 9:**
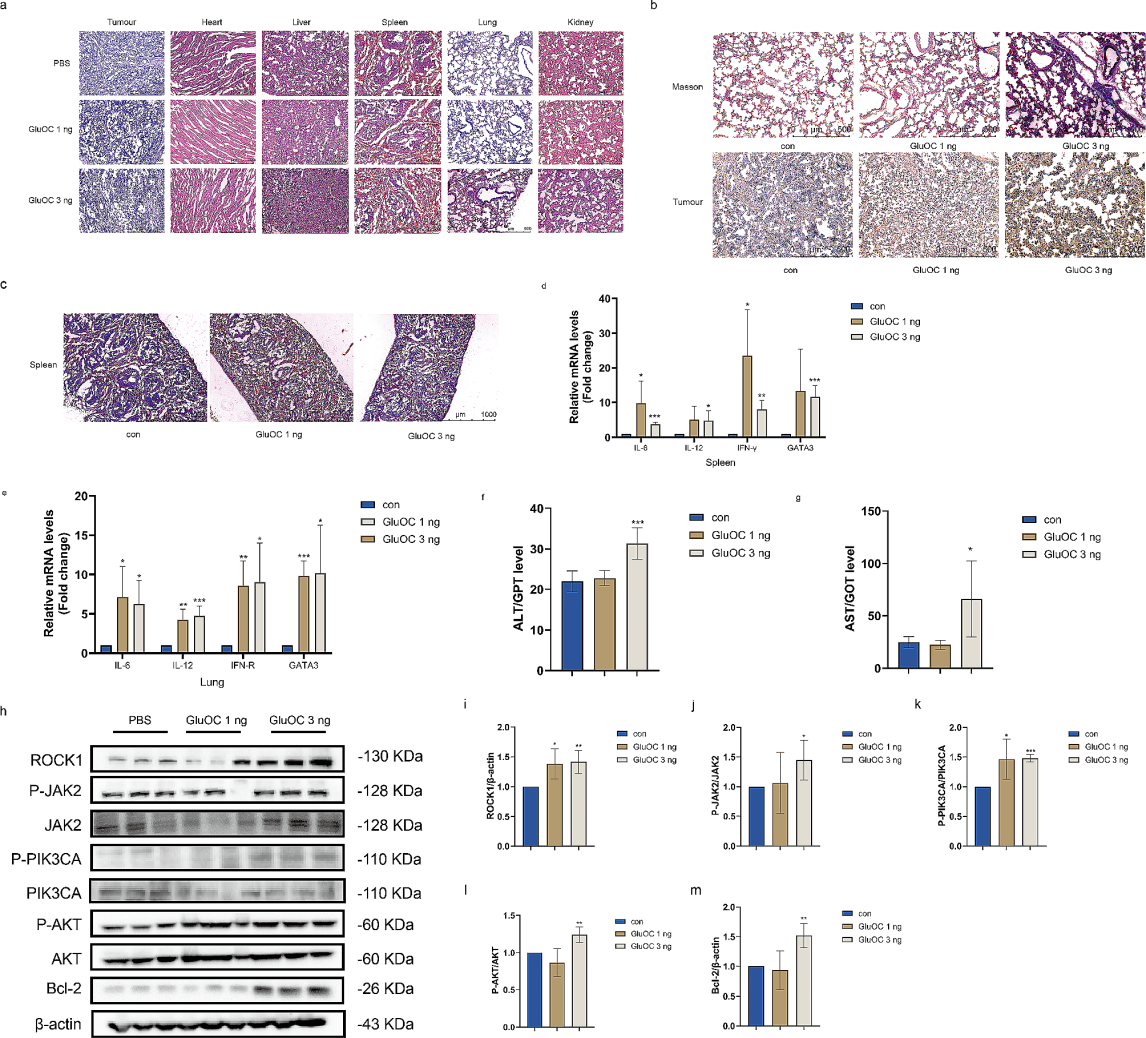
(**a**) HE staining of the tumours and organs of tumour-bearing mice from each group after treatment. (**b**) Masson and Ki67 immunohistochemistry were used to detect lung injury and proliferating cells in tumour sections. (**c**) HE staining of the spleen from each group after treatment (5x). (**d**) The gene expression levels of IL-12, IL-6 IFN-γ and Gata3 in the spleen before and after GluOC were determined by qRT–PCR (*n* = 4). (**e**) The gene expression levels of IL-12, IL-6 IFN-γ and Gata3 in the lungs before and after GluOC were determined by qRT–PCR (*n* = 4). (**f**) Serum ALT/GPT levels in mice (*n* = 6). (**g**) Serum AST/GOT levels in mice (*n* = 6). (**h**-**m**) The relative protein levels in the tumour tissue were statistically analysed by western blotting and quantified densitometrically with ImageJ software. β-Actin was used as the control. The results represent at least three independent experiments, and the data are presented as the mean ± SD (*n* = 4). ^*^p < 0.05, ^**^p < 0.01, ^***^p < 0.001 compared with the PBS group

HE staining of the collected heart, liver, spleen, lung, kidney and tumour tissues revealed metastatic nodules in the lungs and thickened alveolar walls in the 3 ng GluOC group (Fig. 9a). To verify the protein expression of Ki67, immunohistochemistry (IHC) analysis of tumour tissues was performed. Consistent with our previous results, IHC analysis revealed that Ki67 protein expression in tumour tissues treated with 3 ng GluOC was significantly greater than that in the PBS group and 1 ng GluOC group (Fig. 9b). The results indicated that 3 ng of GluOC promoted the proliferation of TNBC cells. In addition, the distinction between red pulp and white pulp in the spleen tissues of mice in the PBS group was clear and the cells were closely arranged, while the distinction between red pulp and white pulp in the spleen tissues of mice in the 3 ng GluOC group was unclear and the margin area was widened (Fig. 9c). The qRT–PCR results showed that the levels of the proinflammatory cytokines IL-12, IL-6, IFN-γ and Gata3 were elevated in the spleen, suggesting inflammation of the spleen (Fig. 9d). There were no obvious pathological changes in the heart or kidney organs. qRT–PCR and Masson’s trichrome staining revesled that the expression of proinflammatory cytokines in the lungs of the mice increased with increasing GluOC dose, and the lungs of the mice showed fibrosis after the administration of 3 ng GluOC (Fig. 9e). In addition, we detected the levels of alanine aminotransferase (ALT) and aspartate aminotransferase (AST) in the serum of the mice, and the results showed that after 3 ng of GluOC treatment, the ALT and AST levels in the serum were greater than those in the control group, indicating hepatocyte injury (Fig. 9f and g). To further determine the role of GluOC in TNBC metastasis, western blotting was performed on collected tumour tissues, and the analysis confirmed that the 1 ng of GluOC and 3 ng of GluOC significantly promoted the expression of ROCK1 and P-PIK3CA in breast cancer. GluOC (3 ng) also significantly promoted the expression of the P-JAK2, P-AKT and Bcl-2 proteins. Together, these results suggest that the ability of GluOC to accelerate TNBC proliferation and metastasis is critical (Fig. 9h-m).

## Discussion

**Fig. 10:**
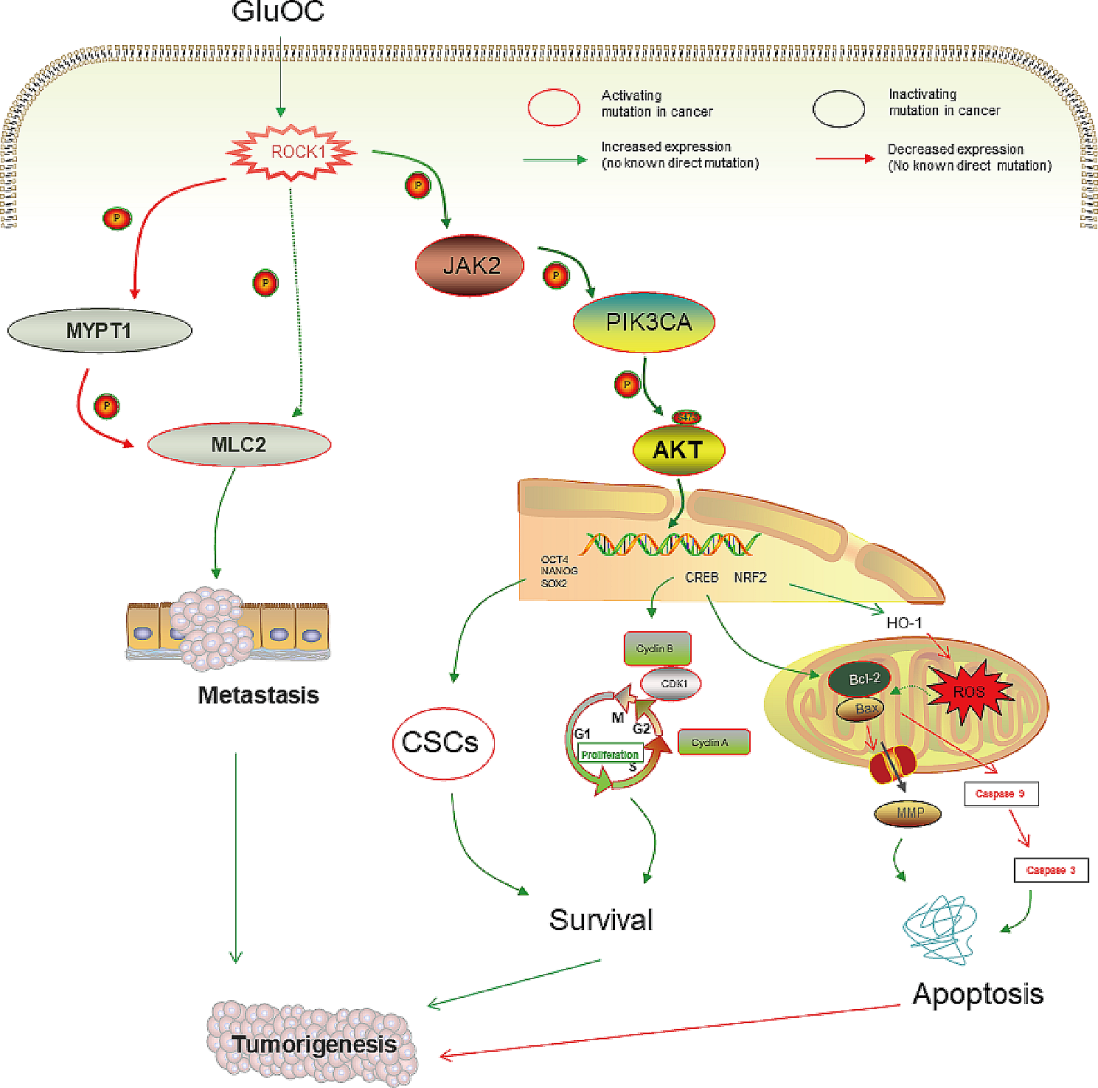
Molecular mechanism by which GluOC promotes the proliferation and migration of MDA-MB-231 breast cancer cells

Overall, our study showed that GluOC promotes the proliferation and metastasis of MDA-MB-231 breast cancer cells through ROCK1. Through in vivo experiments, we found that GluOC promotes TNBC cell proliferation and lung metastasis. These results provide new ideas for the treatment of TNBC and more details regarding its pathogenesis. (Fig. 10).

The treatment of TNBC remains the most challenging among breast tumours due to its high heterogeneity, poor prognosis, and risk of recurrence and metastasis [33]. Hanahan and Weinberg defined the activation of invasion and metastasis as typical features of cancer, where the regulation of the actin-myosin cytoskeleton and multiple changes in tissues play important roles, ultimately leading to significant changes in cell adhesion and movement [34]. Rho-kinase is a serine/threonine kinase that contains ROCK2 and ROCK1. ROCK1 directly or indirectly regulates MLC and MYPT1, thereby causing actin-myosin contraction and regulating cancer cell movement [35]. Through genome-wide transcriptomic analysis, we found that the expression of the ROCK1 gene significantly increased after GluOC treatment. In our study, GluOC activated the ROCK1/MYPT1/MLC2 signalling pathway by promoting the expression of ROCK1, thereby stimulating the migration of cancer cells.

Early changes in cell apoptosis are related to mitochondria, which are mediated by members of the antiapoptotic Bcl-2 protein family and the proapoptotic Bax protein family, as confirmed by the decrease in the apoptosis index of MDA-MB-231 breast cancer cells in this study [36]. The apoptosis process is regulated by Bcl-2 family proteins, which activate caspase proteins that breakdown cells, thereby triggering apoptosis [37]. In this study, GluOC treatment downregulated the protein expression of Bax, caspase 3 and caspase 9 and upregulated the protein expression of the antiapoptotic protein Bcl-2 family, while a ROCK1 inhibitor reversed these effects. GluOC mitigated the reversal effect of ROCK1 inhibitors. These results suggest that GluOC inhibits the apoptosis of MDA-MB-231 breast cancer cells through ROCK1.

The cell cycle is divided into four phases: the G1 phase (prereplication, gap between the end of M phase and S phase), S phase (replication phase, DNA synthesis phase), G2 phase (late replication, gap between the end of S phase and M phase), and M phase (mitosis phase) [38]. The G1/S phase and G2/M phase checkpoints are critical to the cell cycle and can respond to disruption or injury by blocking the cell cycle [39]. However, in tumour cells, G1/S checkpoint regulators often fail, leaving tumour cells prone to enter the S phase [40]. GSEA of whole transcriptome data, and GO and KEGG analyses revealed that the cell cycle progression of MDA-MB-231 breast cancer cells accelerated after GluOC treatment. Subsequent flow cytometry analysis revealed that GluOC alleviated G1–phase arrest induced by a ROCK1 inhibitor (Y-27632), and the results were consistent at both the gene and protein levels. After GluOC treatment, cyclin A2, cyclin B1 and CDK1 increased, while Y-27632 suppressed cell cycle progression in cancer cells. When GluOC and Y-27632 were added simultaneously, the effect of the inhibitor was reversed and cell proliferation was promoted.

The production of ROS is crucial for pro-apoptotic activity, and ROS regulation affects the fate of cancer cells [41]. Studies have shown that the MMP can reflect mitochondrial function, which regulates ATP production, the cell proliferation rate and the cell death cycle [42]. The loss of mitochondrial membrane potential causes mitochondrial dysfunction, which is closely related to the process of apoptosis [43]. Our results showed that GluOC enhanced the antioxidant capacity of cancer cells by promoting Nrf2 and HO-1, but Y-27632 increased the MMP and decreased the mitochondrial membrane potential of cancer cells, thus inducing apoptosis. The addition of GluOC alleviated the cell damage caused by Y-27632 in cancer cells.

Tumour stem cells are cells with self-renewal and multidirectional differentiation potential in tumour tissues [44]. Although they represent a small percentage of cells in tumour tissue, they are highly tumourigenic and have a unique ability to self-regenerate [45]. During the whole process of tumour development, tumour stem cells exhibit strong drug resistance and radiation resistance and have the ability to migrate [38]. Therefore, tumour stem cells are more difficult to eliminate than are ordinary tumour cells [46]. Through transcriptomic analysis, we found that cancer cell stemness plays an important role in the cancer-promoting effect of GluOC. Therefore, we detected the expression levels of the stem genes OCT4, NANOG and SOX2. Consistent with the genomic results, we found that GluOC promoted the protein expression of OCT4, NANOG, and SOX2, while Y-27632 attenuated this effect, but the addition of GluOC significantly alleviated the effect of the inhibitor and induced the proliferation of cancer cells.

We established a tumour-bearing mouse model and injected precultured MDA-MB-231 breast cancer cells into the right abdomen of BALB/c nude mice. Compared with that in the control group, the serum osteocalcin level in the 3 ng GluOC group was significantly greater, which was consistent with the results of previous study [19]. HE staining revealed that 3 ng GluOC had a significant effect on the proliferation of breast tumours and the formation of lung metastases, and promoted the malignancy of MDA-MB-231 cells. In addition, we observed increased expression of inflammatory factors such as IL-6, IL-12, IFN-γ, and Gata 3 in lung and spleen tissues, confirming the formation of an inflammatory microenvironment. In addition, GluOC increased the percentage of cells with positive expression of Ki67 in tumour tissues, which is an important indicator of prognosis in breast cancer patients.

## Conclusion

In summary, our in vitro data suggest that GluOC accelerates the metastasis of MDA-MB-231 breast cancer cells by promoting ROCK1/MYPT1/MLC2 signalling, and by promoting breast cancer cell proliferation via the ROCK1/JAK2/PIK3CA/AKT signalling pathway. Our in vivo results confirmed that 3 ng of GluOC increases the serum osteocalcin concentration in mice, promotes the lung metastasis of breast cancer, and induces inflammatory responses, which are important drivers of TNBC aggressiveness. The in vitro and in vivo results indicate that GluOC plays a role in promoting the malignant behaviour of tumour cells. Based on the GluOC data, this study provides a new therapeutic strategy for controlling TNBC aggression.

### Electronic supplementary material

Below is the link to the electronic supplementary material.

Supplementary Material 1

## Data Availability

No datasets were generated or analysed during the current study.

## Abbreviations

TNBC: Triple-negative breast cancer
PR: Progesterone receptors
ER: Estrogen receptor
HER2: Epidermal growth factor receptor 2
GluOC: Uncarboxylated osteocalcin
GPRC6A: Group C6 member A of the G protein-coupled receptor family
ROCK: Rho-associated coil kinase
DEGs: Differentially expressed genes
OS: Overall survival
MYTP1: Myosin phosphase
MLC: Myosin light chain
MMP: Mitochondrial membrane potential
GSEA: Gene set enrichment analysis
CSCs: Cancer stem cells
ALT: Alanine aminotransferase
AST: Aspartate aminotransferase

## References

[1] Obidiro O, Battogtokh G, Akala EO. Triple negative breast Cancer Treatment options and limitations: Future Outlook. Pharmaceutics 2023, 15(7). 10.3390/pharmaceutics15071796 PMC10384267 37513983

[2] Andreopoulou E, Schweber SJ, Sparano JA, McDaid HM. Therapies for triple negative breast cancer. Expert Opin Pharmacother. 2015;16(7):983–98. 25881743 10.1517/14656566.2015.1032246 PMC5995333

[3] Yao H, He G, Yan S, Chen C, Song L, Rosol TJ, Deng X. Triple-negative breast cancer: is there a treatment on the horizon? Oncotarget. 2017;8(1):1913–24. 27765921 10.18632/oncotarget.12284 PMC5352107

[4] Johnson J, Chow Z, Lee E, Weiss HL, Evers BM, Rychahou P. Role of AMPK and Akt in triple negative breast cancer lung colonization. Neoplasia. 2021;23(4):429–38. 33839456 10.1016/j.neo.2021.03.005 PMC8042649

[5] Vagia E, Mahalingam D, Cristofanilli M. The Landscape of targeted therapies in TNBC. Cancers (Basel) 2020, 12(4). 10.3390/cancers12040916 PMC7226210 32276534

[6] Cortes J, Haiderali A, Huang M, Pan W, Schmid P, Akers KG, Park JE, Frederickson AM, Fasching PA, O’Shaughnessy J. Neoadjuvant immunotherapy and chemotherapy regimens for the treatment of high-risk, early-stage triple-negative breast cancer: a systematic review and network meta-analysis. BMC Cancer. 2023;23(1):792. 37612624 10.1186/s12885-023-11293-4 PMC10463750

[7] Li Z, Yang HY, Zhang XL, Zhang X, Huang YZ, Dai XY, Shi L, Zhou GR, Wei JF, Ding Q. Kinesin family member 23, regulated by FOXM1, promotes triple negative breast cancer progression via activating Wnt/beta-catenin pathway. J Exp Clin Cancer Res. 2022;41(1):168. 35524313 10.1186/s13046-022-02373-7 PMC9077852

[8] Swain SM, Kim SB, Cortes J, Ro J, Semiglazov V, Campone M, Ciruelos E, Ferrero JM, Schneeweiss A, Knott A, et al. Pertuzumab, trastuzumab, and docetaxel for HER2-positive metastatic breast cancer (CLEOPATRA study): overall survival results from a randomised, double-blind, placebo-controlled, phase 3 study. Lancet Oncol. 2013;14(6):461–71. 23602601 10.1016/S1470-2045(13)70130-X PMC4076842

[9] Martiniakova M, Mondockova V, Biro R, Kovacova V, Babikova M, Zemanova N, Ciernikova S, Omelka R. The link between bone-derived factors osteocalcin, fibroblast growth factor 23, sclerostin, lipocalin 2 and tumour bone metastasis. Front Endocrinol (Lausanne). 2023;14:1113547. 36926025 10.3389/fendo.2023.1113547 PMC10012867

[10] Willems BA, Vermeer C, Reutelingsperger CP, Schurgers LJ. The realm of vitamin K dependent proteins: shifting from coagulation toward calcification. Mol Nutr Food Res. 2014;58(8):1620–35. 24668744 10.1002/mnfr.201300743

[11] Han Y, You X, Xing W, Zhang Z, Zou W. Paracrine and endocrine actions of bone-the functions of secretory proteins from osteoblasts, osteocytes, and osteoclasts. Bone Res. 2018;6:16. 29844945 10.1038/s41413-018-0019-6 PMC5967329

[12] Diaz-Franco MC, Franco-Diaz de Leon R, Villafan-Bernal JR. Osteocalcin–GPRC6A: an update of its clinical and biological multi–organic interactions (review). Mol Med Rep. 2019;19(1):15–22. 30431093 10.3892/mmr.2018.9627 PMC6297736

[13] Manolagas SC. Osteocalcin promotes bone mineralization but is not a hormone. PLoS Genet. 2020;16(6):e1008714. 32484816 10.1371/journal.pgen.1008714 PMC7266291

[14] Moser SC, van der Eerden BCJ. Osteocalcin-A versatile bone-derived hormone. Front Endocrinol (Lausanne). 2018;9:794. 30687236 10.3389/fendo.2018.00794 PMC6335246

[15] Ye R, Pi M, Cox JV, Nishimoto SK, Quarles LD. CRISPR/Cas9 targeting of GPRC6A suppresses prostate cancer tumourigenesis in a human xenograft model. J Exp Clin Cancer Res. 2017;36(1):90. 28659174 10.1186/s13046-017-0561-x PMC5490090

[16] Kayed H, Bekasi S, Keleg S, Michalski CW, Giese T, Friess H, Kleeff J. BGLAP is expressed in pancreatic cancer cells and increases their growth and invasion. Mol Cancer. 2007;6:83. 18163903 10.1186/1476-4598-6-83 PMC2245975

[17] Nimptsch K, Rohrmann S, Nieters A, Linseisen J. Serum undercarboxylated osteocalcin as biomarker of vitamin K intake and risk of prostate cancer: a nested case-control study in the Heidelberg cohort of the European prospective investigation into cancer and nutrition. Cancer Epidemiol Biomarkers Prev. 2009;18(1):49–56. 19124480 10.1158/1055-9965.EPI-08-0554

[18] Engblom C, Pfirschke C, Zilionis R, Da Silva Martins J, Bos SA, Courties G, Rickelt S, Severe N, Baryawno N, Faget J et al. Osteoblasts remotely supply lung tumours with cancer-promoting SiglecF(high) neutrophils. *Science* 2017, 358(6367). 10.1126/science.aal5081 PMC6343476 29191879

[19] Pietschmann P, Zielinski C, Woloszczuk W. Serum osteocalcin levels in breast cancer patients. J Cancer Res Clin Oncol. 1989;115(5):456–8. 2808485 10.1007/BF00393337 PMC12211687

[20] Xu J, Ma L, Wang D, Yang J. Uncarboxylated osteocalcin promotes proliferation and metastasis of MDA-MB-231 cells through TGF-beta/SMAD3 signaling pathway. BMC Mol Cell Biol. 2022;23(1):18. 35413833 10.1186/s12860-022-00416-7 PMC9003967

[21] Wei L, Surma M, Shi S, Lambert-Cheatham N, Shi J. Novel insights into the roles of rho kinase in Cancer. Arch Immunol Ther Exp (Warsz). 2016;64(4):259–78. 26725045 10.1007/s00005-015-0382-6 PMC4930737

[22] Yan L, Li H, An W, Wei W, Zhang X, Wang L. Mex-3 RNA binding MEX3A promotes the proliferation and migration of breast cancer cells via regulating RhoA/ROCK1/LIMK1 signaling pathway. Bioengineered. 2021;12(1):5850–8. 34486491 10.1080/21655979.2021.1964155 PMC8806898

[23] Xu J, Yang X, Deng Q, Yang C, Wang D, Jiang G, Yao X, He X, Ding J, Qiang J, et al. TEM8 marks neovasculogenic tumour-initiating cells in triple-negative breast cancer. Nat Commun. 2021;12(1):4413. 34285210 10.1038/s41467-021-24703-7 PMC8292527

[24] Retraction for et al. Gilkes., Hypoxia-inducible factors mediate coordinated RhoA-ROCK1 expression and signaling in breast cancer cells. *Proc Natl Acad Sci U S A* 2022, 119(38):e2213288119. 10.1073/pnas.2213288119 PMC9499587 36053739

[25] Luo S, Wang H, Bai L, Chen Y, Chen S, Gao K, Wang H, Wu S, Song H, Ma K, et al. Activation of TMEM16A ca(2+)-activated Cl(-) channels by ROCK1/moesin promotes breast cancer metastasis. J Adv Res. 2021;33:253–64. 34603794 10.1016/j.jare.2021.03.005 PMC8463928

[26] Zhu S, Wu Y, Song B, Yi M, Yan Y, Mei Q, Wu K. Recent advances in targeted strategies for triple-negative breast cancer. J Hematol Oncol. 2023;16(1):100. 37641116 10.1186/s13045-023-01497-3 PMC10464091

[27] O’Shea JJ, Schwartz DM, Villarino AV, Gadina M, McInnes IB, Laurence A. The JAK-STAT pathway: impact on human disease and therapeutic intervention. Annu Rev Med. 2015;66:311–28. 25587654 10.1146/annurev-med-051113-024537 PMC5634336

[28] Nusse R, Clevers H. Wnt/beta-Catenin signaling, Disease, and emerging therapeutic modalities. Cell. 2017;169(6):985–99. 28575679 10.1016/j.cell.2017.05.016

[29] Briscoe J, Therond PP. The mechanisms of hedgehog signalling and its roles in development and disease. Nat Rev Mol Cell Biol. 2013;14(7):416–29. 23719536 10.1038/nrm3598

[30] Johnson J, Chow Z, Napier D, Lee E, Weiss HL, Evers BM, Rychahou P. Targeting PI3K and AMPKalpha Signaling alone or in combination to Enhance Radiosensitivity of Triple negative breast Cancer. Cells 2020, 9(5). 10.3390/cells9051253 PMC7291172 32438621

[31] Kriegsmann M, Endris V, Wolf T, Pfarr N, Stenzinger A, Loibl S, Denkert C, Schneeweiss A, Budczies J, Sinn P, et al. Mutational profiles in triple-negative breast cancer defined by ultradeep multigene sequencing show high rates of PI3K pathway alterations and clinically relevant entity subgroup specific differences. Oncotarget. 2014;5(20):9952–65. 25296970 10.18632/oncotarget.2481 PMC4259450

[32] Cossu-Rocca P, Orru S, Muroni MR, Sanges F, Sotgiu G, Ena S, Pira G, Murgia L, Manca A, Uras MG, et al. Analysis of PIK3CA mutations and activation pathways in Triple negative breast Cancer. PLoS ONE. 2015;10(11):e0141763. 26540293 10.1371/journal.pone.0141763 PMC4634768

[33] Tian Y, Zhang P, Mou Y, Yang W, Zhang J, Li Q, Dou X. Silencing Notch4 promotes tumourigenesis and inhibits metastasis of triple-negative breast cancer via nanog and Cdc42. Cell Death Discov. 2023;9(1):148. 37149651 10.1038/s41420-023-01450-w PMC10164131

[34] Amano M, Nakayama M, Kaibuchi K. Rho-kinase/ROCK: a key regulator of the cytoskeleton and cell polarity. Cytoskeleton (Hoboken). 2010;67(9):545–54. 20803696 10.1002/cm.20472 PMC3038199

[35] Kim S, Kim SA, Han J, Kim IS. Rho-Kinase as a target for Cancer Therapy and its immunotherapeutic potential. Int J Mol Sci 2021, 22(23). 10.3390/ijms222312916 PMC8657458 34884721

[36] Liao M, Qin R, Huang W, Zhu HP, Peng F, Han B, Liu B. Targeting regulated cell death (RCD) with small-molecule compounds in triple-negative breast cancer: a revisited perspective from molecular mechanisms to targeted therapies. J Hematol Oncol. 2022;15(1):44. 35414025 10.1186/s13045-022-01260-0 PMC9006445

[37] Knight T, Luedtke D, Edwards H, Taub JW, Ge Y. A delicate balance - the Bcl-2 family and its role in apoptosis, oncogenesis, and cancer therapeutics. Biochem Pharmacol. 2019;162:250–61. 30668936 10.1016/j.bcp.2019.01.015

[38] Wu Y, Song Y, Wang R, Wang T. Molecular mechanisms of tumour resistance to radiotherapy. Mol Cancer. 2023;22(1):96. 37322433 10.1186/s12943-023-01801-2 PMC10268375

[39] Ghelli Luserna, di Rora A, Iacobucci I, Martinelli G. The cell cycle checkpoint inhibitors in the treatment of leukemias. J Hematol Oncol. 2017;10(1):77. 28356161 10.1186/s13045-017-0443-x PMC5371185

[40] Halazonetis TD, Gorgoulis VG, Bartek J. An oncogene-induced DNA damage model for cancer development. Science. 2008;319(5868):1352–5. 18323444 10.1126/science.1140735

[41] Nakamura H, Takada K. Reactive oxygen species in cancer: current findings and future directions. Cancer Sci. 2021;112(10):3945–52. 34286881 10.1111/cas.15068 PMC8486193

[42] Waseem M, Wang BD. Promising strategy of mPTP modulation in Cancer Therapy: an emerging progress and future insight. Int J Mol Sci 2023, 24(6). 10.3390/ijms24065564 PMC10051994 36982637

[43] Killarney ST, Tait SWG, Green DR, Wood KC. Sublethal engagement of apoptotic pathways in residual cancer. Trends Cell Biol 2023. 10.1016/j.tcb.2023.07.005 PMC10858294 37573235

[44] O’Brien CA, Kreso A, Dick JE. Cancer stem cells in solid tumours: an overview. Semin Radiat Oncol. 2009;19(2):71–7. 19249644 10.1016/j.semradonc.2008.11.001

[45] Batlle E, Clevers H. Cancer stem cells revisited. Nat Med. 2017;23(10):1124–34. 28985214 10.1038/nm.4409

[46] Chang JC. Cancer stem cells: role in tumour growth, recurrence, metastasis, and treatment resistance. Med (Baltim). 2016;95(1 Suppl 1):S20–5. 10.1097/MD.0000000000004766 PMC5599212 27611935

