# Supplementary material for: GluOC promotes proliferation and metastasis of TNBC through the ROCK1 signaling pathway
Source: Cancer Cell Int. 2024 Jul 25;24:263. doi: 10.1186/s12935-024-03445-8 (PMC11270849; doi:10.1186/s12935-024-03445-8)
Supplement: Supplementary file 1 — Supplementary Material 1 [file 12935_2024_3445_MOESM1_ESM.docx]

**GluOC promotes proliferation and metastasis of TNBC through the ROCK1 signaling pathway**

Jiaojiao Xu^1^, Keting Dong^1^, Xue Bai^1^, Miao Zhang^1^, Qian Du^1^, Lei Chen^1^ and Jianhong Yang^1*^

^1^Medical School, University of Chinese Academy of Sciences, Beijing 100049, P.R. China

*Correspondence to: Professor Jianhong Yang, Medical School, University of Chinese Academy of Sciences, 19A Yuquan Road, Beijing 100049, P.R. China

E-mail: [yangjh@ucas.edu.cn](mailto:yangjh@ucas.edu.cn)

**Supplementary method 1**

Total RNA was extracted from the 10 pairs of OSCC tissues and para-carcinoma tissues using TRIzol® Reagent according the manufacturer’s instructions (Magen). Paired-end libraries were prepared using a ABclonal mRNA-seq Lib Prep Kit (ABclonal, China) following the manufacturer's instructions. Sequencing was performed with an Illumina Novasea 6000 /MGISEO-T7 instrument. The data generated from Illumina/BGl platform were used for bioinformatics analysis. Then clean reads were separately aligned to reference genome with orientation mode using HISAT2 software (http://dachwankimlab.github.io/hisat2) to obtain mapped reads. Feature Counts (http://subread.sourceforge.net/) was used to count the reads numbers mapped to each gene. And then FPKM of each gene was calculated based on the length of the gene and reads count mapped to this gene. Differential expression analysis was performed using the DESeq2 (<http://bioconductor.org/packages/release/bioc/html/DESeq2.htm>), Differential expression genes (DEGs) with |log_2_FC| > l and *P*-adj < 0.05 were considered to be significantly different expressed genes. The Gene Ontology (GO) and Kyoto Encyclopedia of Genes and Genomes (KEGG) enrichment analysis of differential genes can explain the functional enrichment of differential genes and clarify the differences between samples at the gene function level. ClusterProfiler R software package was used to GO function enrichment and KEGG pathway enrichment analysis. When *p*-value < 0.05, it is considered that the GO or KEGG function is significantly enriched.

**Supplemental Table**

Table 1 The quantitative real-time PCR primer information.

| Gene | Primer sequences（5’→3’） |
| --- | --- |
| Human-RhoA | F: GGAAAGCAGGTAGAGTTGGCT  R: GGCTGTCGATGGAAAAACACAT |
| ROCK1 | F: AAGTGAGGTTAGGGCGAAATG  R: AAGGTAGTTGATTGCCAACGAA |
| Human-CREB | F: CCCAGCCATCAGTTATTCAG  R: GAGTTGGCACCGTTACAGTG |
| Human-Bax | F: TCTGACCCTAGCTCTTTCCT  R: CACCTGTAATCCCAGCACTT |
| Human-Bcl-2 | F: TAGTGTGTATGCCCTGCTTTC  R: CCTCTGTGATGCTGAAAGGTTA |
| Human-PIK3CA | F: CGAGTGGTTGGGCAATGAAA  R: AATGCTTTACTTCGCCGTCC |
| Human-JAK2 | F: TAGATGAGTCAACCAGGCATAATG  R: CCGCCACTGAGCAAAGAG |
| Human-Cyclin A | F: GCAAACAGTAAACAGCCTGCGTTC  R: TGGGTCCAGGTAAACTAATGGCTG |
| Human-Cyclin B | F: GGAGAGGTTGATGTCGAGCAAC  R: GAGAAGGAGGAAAGTGCACCATG |
| Human-CDK1 | F: AGTCTTCAGGATGTGCTTATGCAG  R: AGAATCCATGTACTGACCAGGAGG |
| Human-SOX2 | F: AGCTACAGCATGATGCAGGA  R: GGTCATGGAGTTGTACTGCA |
| Human-OCT4 | F: AGCGAACCAGTATCGAGAAC  R: TTACAGAACCACACTCGGAC |
| Human-NANOG | F: ACAACTGGCCGAAGAATAGCA  R: GGTTCCCAGTCGGGTTCAC |
| Human-β-actin | F: AGATGTGGTCAGCAAGCAG  R: GCGCAAGTTAGGTTTTGTCA |
| Mus-IL-12 | F: TGGTTTGCCATCGTTTTGCTG  R: TGGTTTGCCATCGTTTTGCTG |
| Mus-IL-6 | F: CTGCAAGAGACTTCCATCCAG  R: AGTGGTATAGACAGGTCTGTTGG |
| Mus-Ifnγ | F: AAGCGTCATTGAATCACACCTG  R: TGACCTCAAACTTGGCAATACTC |
| Mus-Gata3 | F: CTCGGCCATTCGTACATGGAA  R: GGATACCTCTGCACCGTAGC |
| Mus-β-actin | F: GATCTGGCACCACACCTTCT  R: GGGGTGTTGAAGGTCTCAAA |

**Supplemental information**


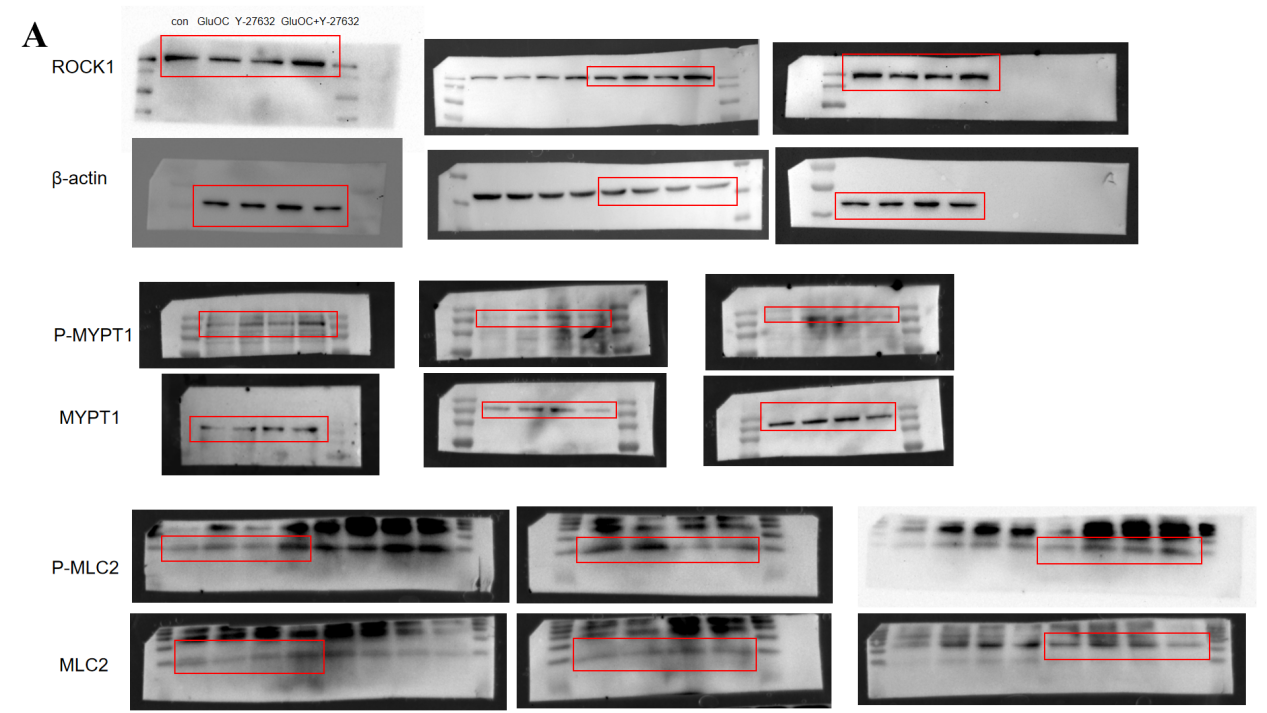


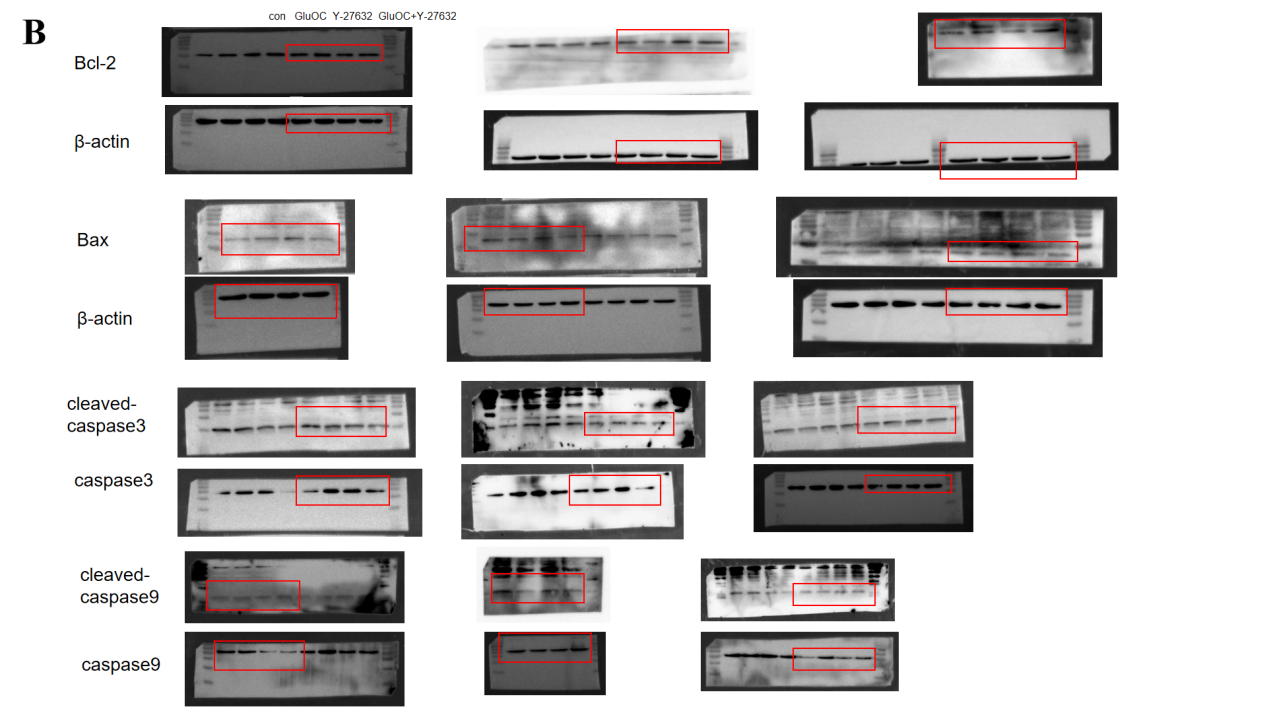
**Figure S1** Western blot full size image of MDA-MB-231 cells. Figure **A** shows the MDA-MB-231 cells were grouped into con, 160 ng GluOC, Y-27632 and GluOC+Y-27632. ROCK1, P-MYPT1, MYPT1, P-MLC, and full-size western blot images of MLC were used to evaluate GluOC's increased migration of MDA-MB-231 cells. As shown in figure **B**, GluOC inhibited apoptosis of MDA-MB-231 cells after added GluOC. Full-size western blot images of Bcl-2, Bax, cleaved caspase9, caspase9, cleaved caspase3, caspase3. The red box indicates the area of grouping and calculation.


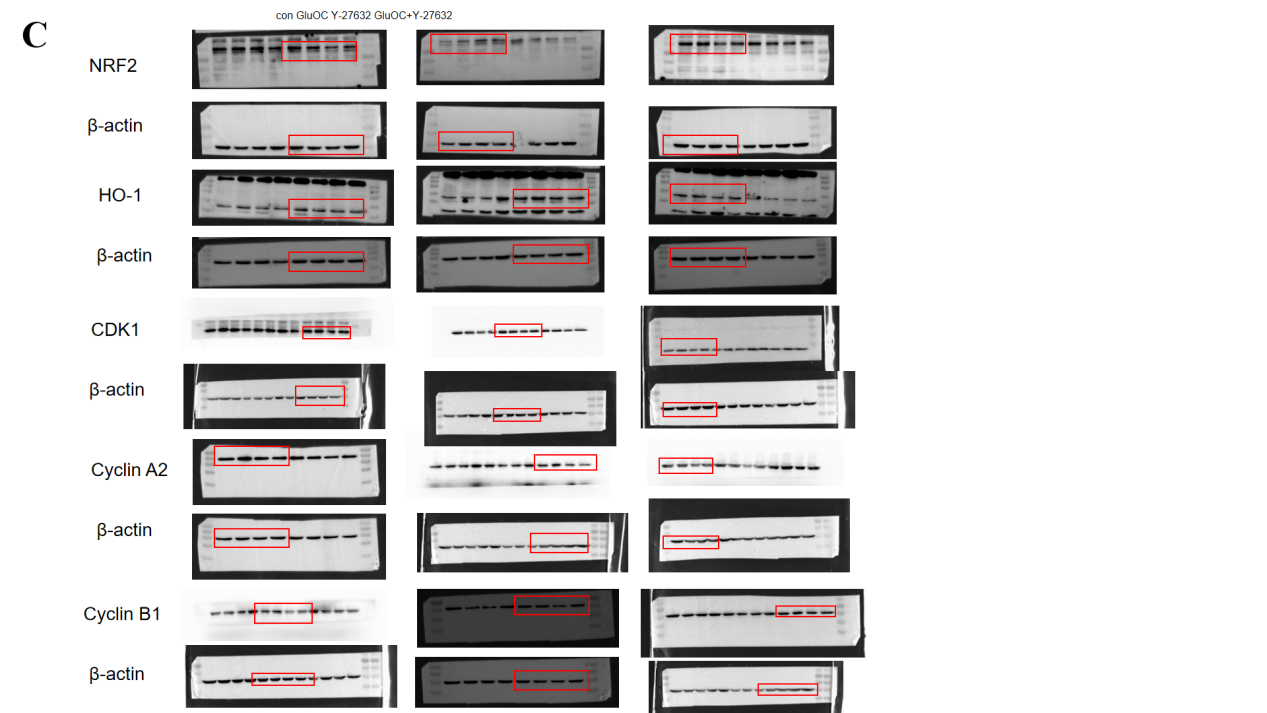


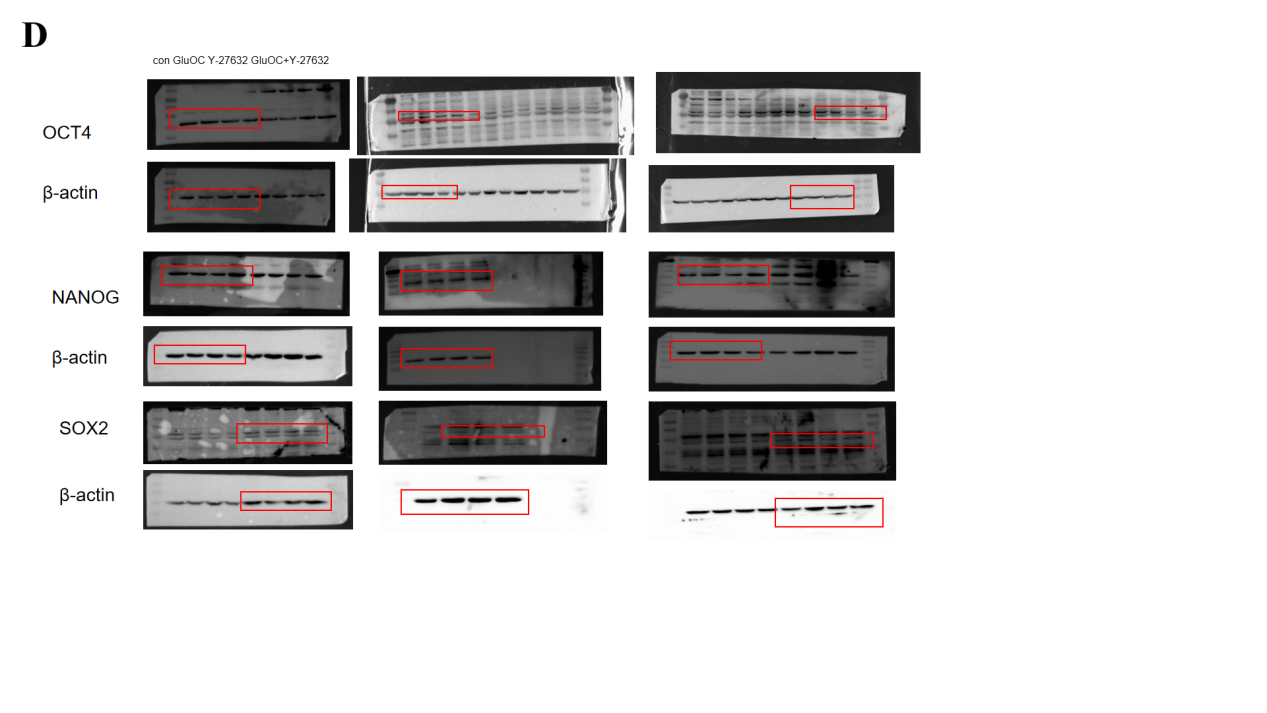
**Figure S2** Western blot full size image of MDA-MB-231 cells. Figure **C** is NRF2, HO-1,CDK1, Cyclin A2, and full-size western of Cyclin B1 in MDA-MB-231 cells. Figure **D** is OCT4, NANOG, and full-size western of SOX2.It is divided into four components: con,160ng GluOC, Y-27632, GluOC+Y-27632, then the blot images were used to assess GluOC's Inhibits oxidative stress of cells, promotes cell cycle progression of cancer cells and enhances the expression of stemness gene. The red box of a full-size western blot image used for grouping and calculation.


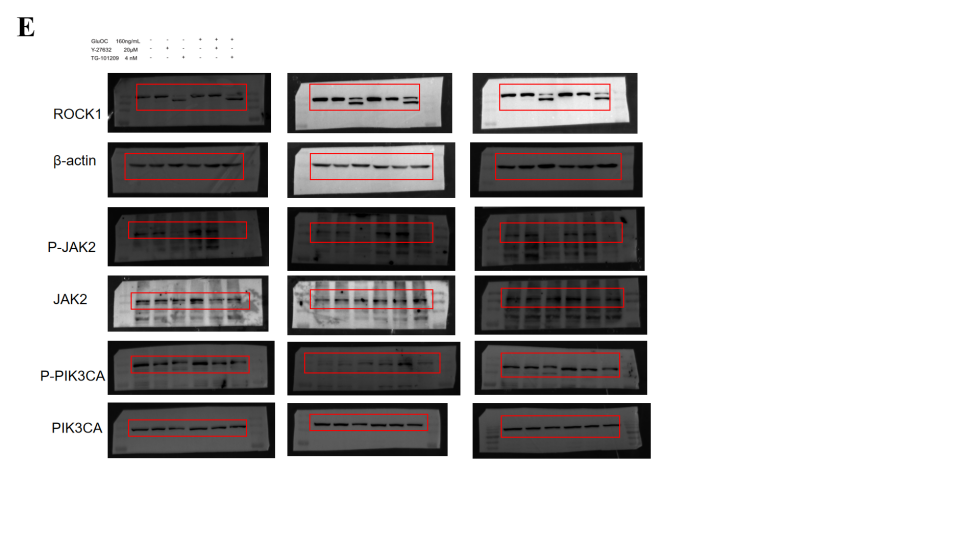

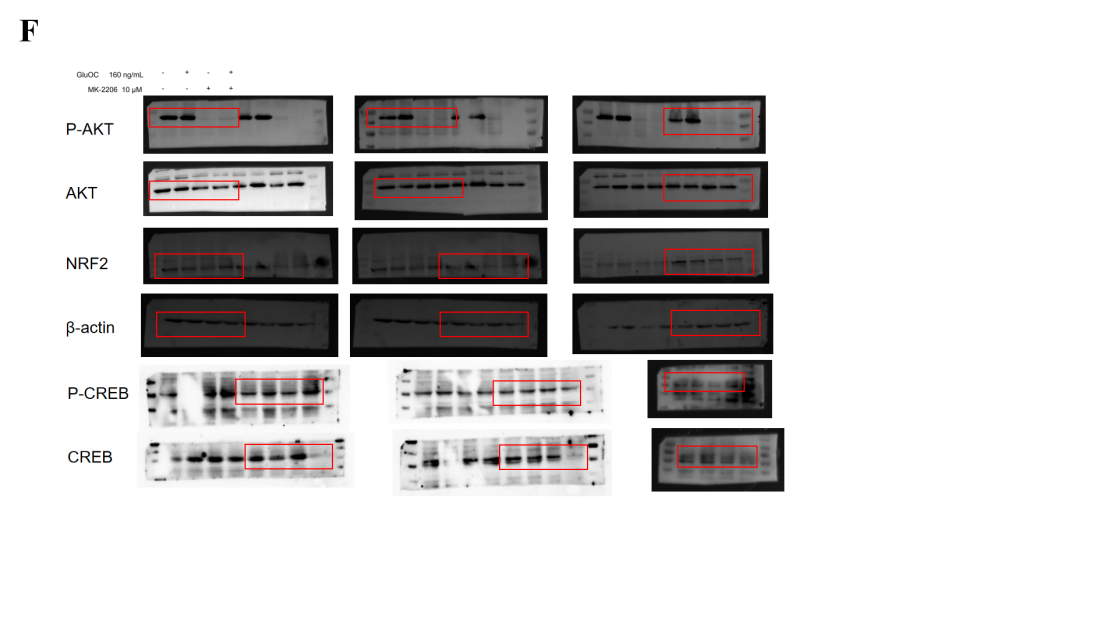


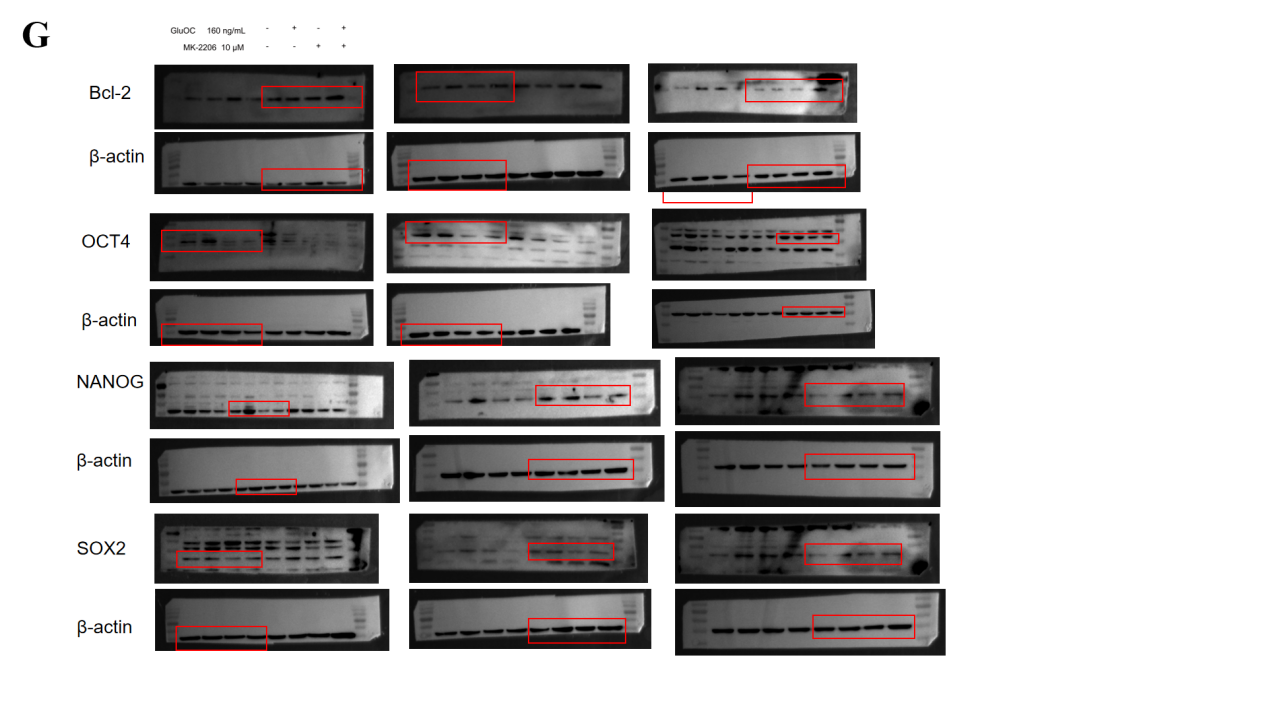


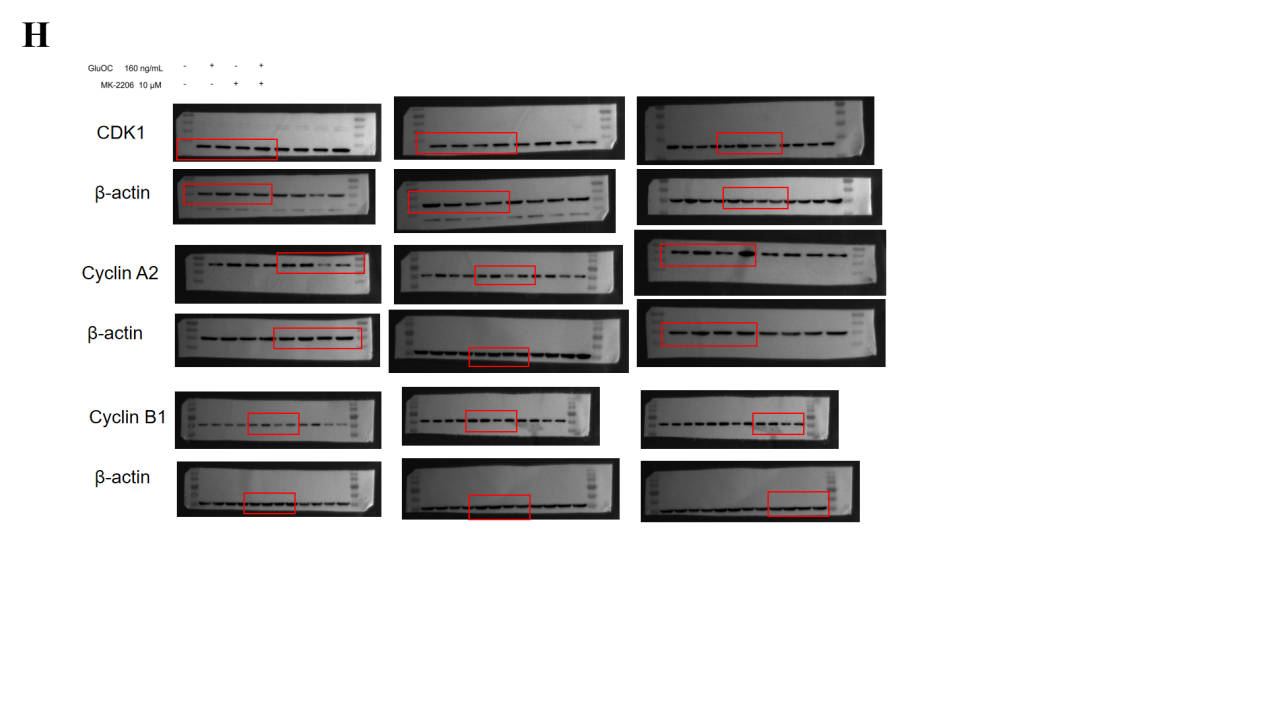


**Figure S3** Full-size Western blot image of MDA-MB-231 cells. Figure **E** shows full-size western blot images of ROCK1, P-JAK2, JAK2, P-PIK3CA, and PIK3CA in MDA-MB-231 cells after addition of ROCK1 inhibitor, JAK2 phosphorylation inhibitor, and GluOC to verify the signaling pathway. As shown in figure **F**, we detected the expression levels of NRF2, P-CREB and CREB proteins in cancer cells after adding AKT phosphorylation inhibitors. Figure G and H shows the expression of proliferating protein Bcl-2 and cyclins CDK1, Cyclin A2 and Cyclin B1 after adding AKT phosphorylation inhibitor. In addition, the expression levels of OCT4, NANOG and SOX2 proteins related to stem cell properties were also detected. The red box of a full-size western blot image is the area for grouping calculations.


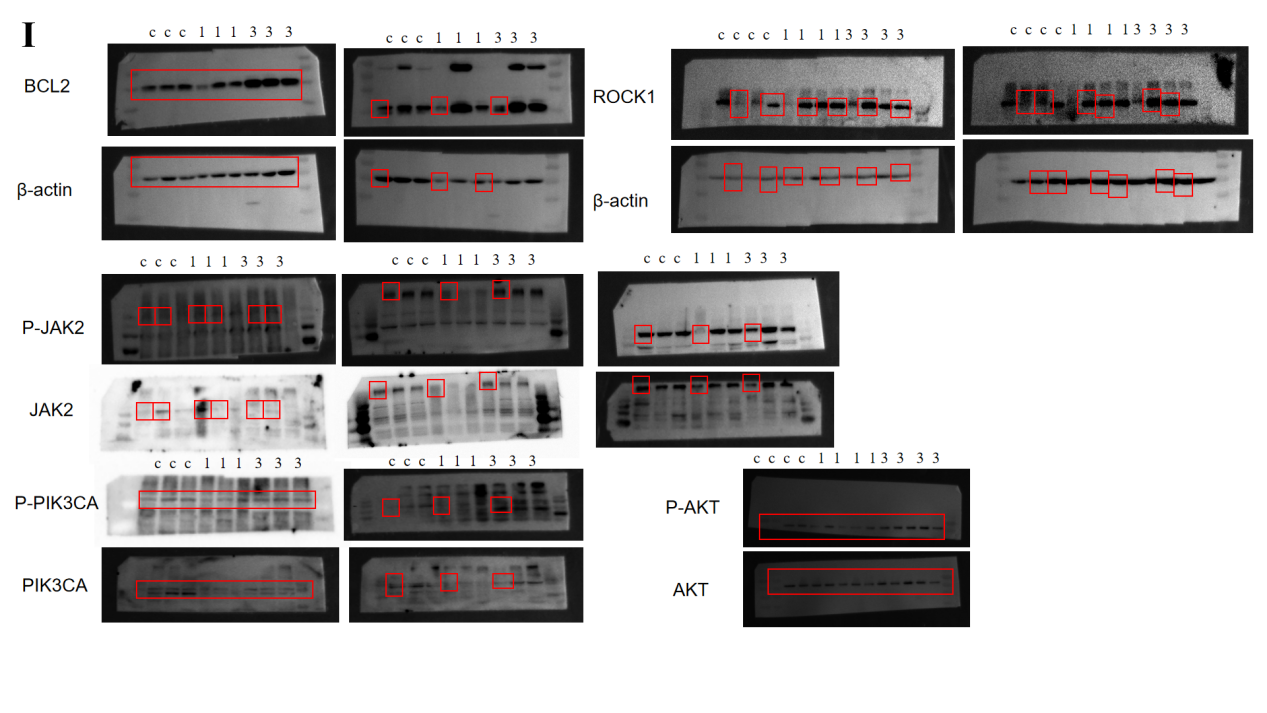


**Figure S4** Full-size images of western blots of tumor. As shown in figure **I**, tumor-bearing mice were divided into three components, PBS,1ng GluOC and 3ng GluOC, showing the full-size western blot images of animal tumor tissue. Here is the expression level of ROCK1, P-JAK2, P-PIK3CA, P-AKT and Bcl-2 proteins in tumor tissue, where c=con, 1=1ng GluOC, 3=3ng GluOC.The red boxes indicate the cropped regions.

In the process of electrical transfer of proteins, multiple proteins were transfected from different parts of the same SDS-PAGE gel using pre-stained protein marker as reference. In this process, we used membrane regeneration solution to incubate different antibodies in PVDF membrane again. We have provided three independent repeats and submitted the whole western blot picture as an attachment. In addition, we labeled the molecular weight of the protein in the figures.
